# PEPN-GRN: A Petri net-based approach for the inference of gene regulatory networks from noisy gene expression data

**DOI:** 10.1371/journal.pone.0251666

**Published:** 2021-05-14

**Authors:** Deepika Vatsa, Sumeet Agarwal

**Affiliations:** Department of Electrical Engineering, Indian Institute of Technology Delhi, New Delhi, India; Pázmány Péter Catholic University, HUNGARY

## Abstract

The inference of gene regulatory networks (GRNs) from expression data is a challenging problem in systems biology. The stochasticity or fluctuations in the biochemical processes that regulate the transcription process poses as one of the major challenges. In this paper, we propose a novel GRN inference approach, named the Probabilistic Extended Petri Net for Gene Regulatory Network (PEPN-GRN), for the inference of gene regulatory networks from noisy expression data. The proposed inference approach makes use of transition of discrete gene expression levels across adjacent time points as different evidence types that relate to the production or decay of genes. The paper examines three variants of the PEPN-GRN method, which mainly differ by the way the scores of network edges are computed using evidence types. The proposed method is evaluated on the benchmark DREAM4 *in silico* data sets and a real time series data set of *E. coli* from the DREAM5 challenge. The PEPN-GRN_v3 variant (the third variant of the PEPN-GRN approach) sought to learn the weights of evidence types in accordance with their contribution to the activation and inhibition gene regulation process. The learned weights help understand the time-shifted and inverted time-shifted relationship between regulator and target gene. Thus, PEPN-GRN_v3, along with the inference of network edges, also provides a functional understanding of the gene regulation process.

## Introduction

There are different kinds of processes in the cell that work together to perform different activities. Depending upon the components and their interaction types, at large, we have three kinds of biochemical networks at the sub-cellular level: metabolic networks, signal transduction networks, and gene regulatory networks. A gene regulatory network (GRN) is a network of gene-gene interactions that govern their expression. Regulation of gene expression is important as it regulates the amount of protein production in the cell.

Inference of gene regulatory networks is considered as one of the key problems in Systems Biology [1, 2]. There has been an exponential growth of reverse engineering methods for network reconstruction over the last two decades [3–7]. Even though, since the last decade, high-throughput techniques like microarrays and RNA-seq made the availability of gene expression data possible, network inference is still considered a challenging problem given the factors like stochasticity, measurement noise, missing expression values, limited time points, etc. in the expression data [8]. Apart from these constraints, noise introduced due to high-throughput techniques like DNA microarrays where the data have a high noise-to-signal ratio also creates a hurdle in the network inference and is often overlooked while developing these reverse engineering methods [9].

Different approaches have been used recently for network inference from time series data such as ODE models [10], Bayesian networks [11], Gaussian graphical models [12], neural networks [13], and information theory methods [14]. Recent reviews of gene regulatory network inference methods and their limitations are given in [15, 16]. Huynh-Thu et al. [15] presented an introductory review of the field by first providing the background of core biological concepts involved, and then introducing different inference approaches in a categorized way. The paper also provides links to the publicly available software tools for network inference. Banf et al. [16] presented a review of GRN inference methods, their limitations, and their application on plant species *A. Thaliana*. The paper also focuses on different data types that can be used for GRN inference such as transcription factor binding site information, chromatin conformation mapping data, and the need to integrate different data sets to obtain more adequate results.

Expression data could be measured at steady-state or generated as a time series. Generally, in time series data generation, the steady-state of the network is perturbed, and the evolving expression values are recorded until the network reaches the steady-state again. Since time series data sets have evolving expression values of genes recorded at different time instants, they are considered to be more informative about the dynamics of the underlying network than steady-state data. Causal relationships among genes can be inferred from time series data only. It has been shown that the inference approach GENIE3 (which is based on steady-state data), performs poorly on time series data [17]. Thus, it suggests that the approach undertaken for the inference process should make use of the information of multiple time points to better capture the interactions between the genes.

In gene regulatory networks [18, 19], genes interact with one another to perform specific cell functions. Gene-gene interactions are regulatory interactions between a regulator gene (also known as a transcription factor (TF)) and a target gene. A target gene is expressed depending on the activity of regulating genes. The interaction from the regulator gene to a target gene can either be activating or inhibiting. Activating regulation accelerates the production of the target gene (and the target gene is expressed) while in inhibiting regulation, it slows down the production of the target gene even less than it was produced (expressed) in the absence of this inhibiting regulating gene. Generally, there is some synthesis constant associated with the production of each target gene. Due to that, a gene can be expressed (though to a very low level) even in the absence of any regulator gene [7, 20]. Similarly, there is some decay constant associated with each gene. However, gene degradation is a slower process than gene synthesis. Thus, in the presence of inhibiting regulation, the net result can be seen as that of the degradation of the target gene [7, 20].

Gene expression data is of the form of continuous gene expression levels. In gene regulation, a regulator gene remains in its inactive form until it reaches a threshold concentration. Only when its concentration increases above the threshold concentration, it becomes active [21]. The sigmoid shape of real interaction in gene regulation can be interpreted as a step function in its logical abstraction [22]. Therefore, the regulatory mechanism of genes can be interpreted in the switch form, that is, ON or OFF. In a logical abstraction view, a regulator gene (or TF) in its ON state attaches to the DNA and initiates the regulation of a target gene. Thus, the target gene upon its production goes from OFF to ON state. The discretization of time series data helps in identifying potential regulator genes for a target gene.

Researchers have tried to explore the behaviour of GRNs using discrete data. Ito et al. [23] qualitatively analyze behaviour of a GRN using the logical abstraction of gene expression levels using two states ON and OFF and exploited the switching between two states to capture the GRN behaviour. Schaub et al. [24] used many discrete levels to represent a gene state and model some aspects of biological systems like negative auto-regulation. Thus, the logical abstraction of the continuous gene expression values preserves the qualitative features of the system dynamics such as oscillatory behaviour, steady-state, and reachability [22]. Küffner et al. [25] proposed a Petri net model with fuzzy logic (PNFL) for GRN inference. The PNFL is a discrete rule-based approach that performs simulations to retrieve system dynamics along with its topology.

In this paper, we will present a discrete system-based approach for the inference of GRNs while dealing with the issues of noise, and stochasticity. A discrete system-based approach means that the approach is based on a discrete system where a countable number of gene expression levels exist in the data.

## Materials and methods

### Data sets

#### DREAM4 *in silico network* challenge data set

DREAM4 *in silico* network challenge provides biologically plausible simulated expression data of 5 networks of size 10-gene and 5 networks of size 100-genes with the aim to recover the underlying network [2, 26, 27]. We used time series data sets of networks from the challenge. The time series data sets were generated by applying a perturbation to the network at time 0 and let the state change happen till time 10, after which the perturbation is removed and the network returns to its original state. Each time series consists of noisy expression data. For 10-gene networks, 5 time series were provided, and for 100-gene networks, 10 time series were provided. Each time series consists of 21 time points.

#### Real gene expression data set of *Escherichia coli*

*E. coli* is a well-studied organism in literature. The *E. coli* data set considered here is retrieved from the DREAM5 network inference challenge [6]. The complete data set of *E. coli* consists of 4511 genes and is generated from 805 microarray chip experiments. We have extracted the data corresponding to the time series experiments. The different time series contains 618 time points in total for 4511 genes. Then, to extract a sub-network, 400 genes are sampled randomly from 4511 genes. In the sub-network of 400 genes, only 236 are the participating genes (i.e., either out- degree > 0 or in- degree > 0 or both) and contains 257 edges. Finally, the time series data corresponding to these 236 genes is extracted. For the experiment, we used this sampled sub-network of 236 genes containing 257 edges and 38 TFs. We have used the known 38 TFs as background knowledge in our inference approach to restrict the edges in the inferred network.

### Petri nets

A Petri Net (PN) [28, 29] is a directed bipartite graph that can be used to model system behaviour. The concept of the Petri net originated from Carl Adam Petri’s dissertation [30] in 1962. It contains two types of nodes: *place* nodes that represent components and *transition* nodes that represent interactions between the components. Each place contains tokens that represent the discretized amount of the component. A particular distribution of tokens over all places represents the *marking* of the net, which reflects the state of the system. Each transition has some input place and some output place. Directed arcs go from input place to transition and from transition to output place. Sufficient tokens in the input place enable the transition to fire and produce tokens at output place. Thus, the input place is considered as a pre-condition which, when satisfied, enables the transition to fire (or event to happen) and produce post-condition in the form of output place. The flow of tokens represents the underlying dynamics of the system, which makes it a visual tool to understand the functioning of a system.

**Fig 1:**
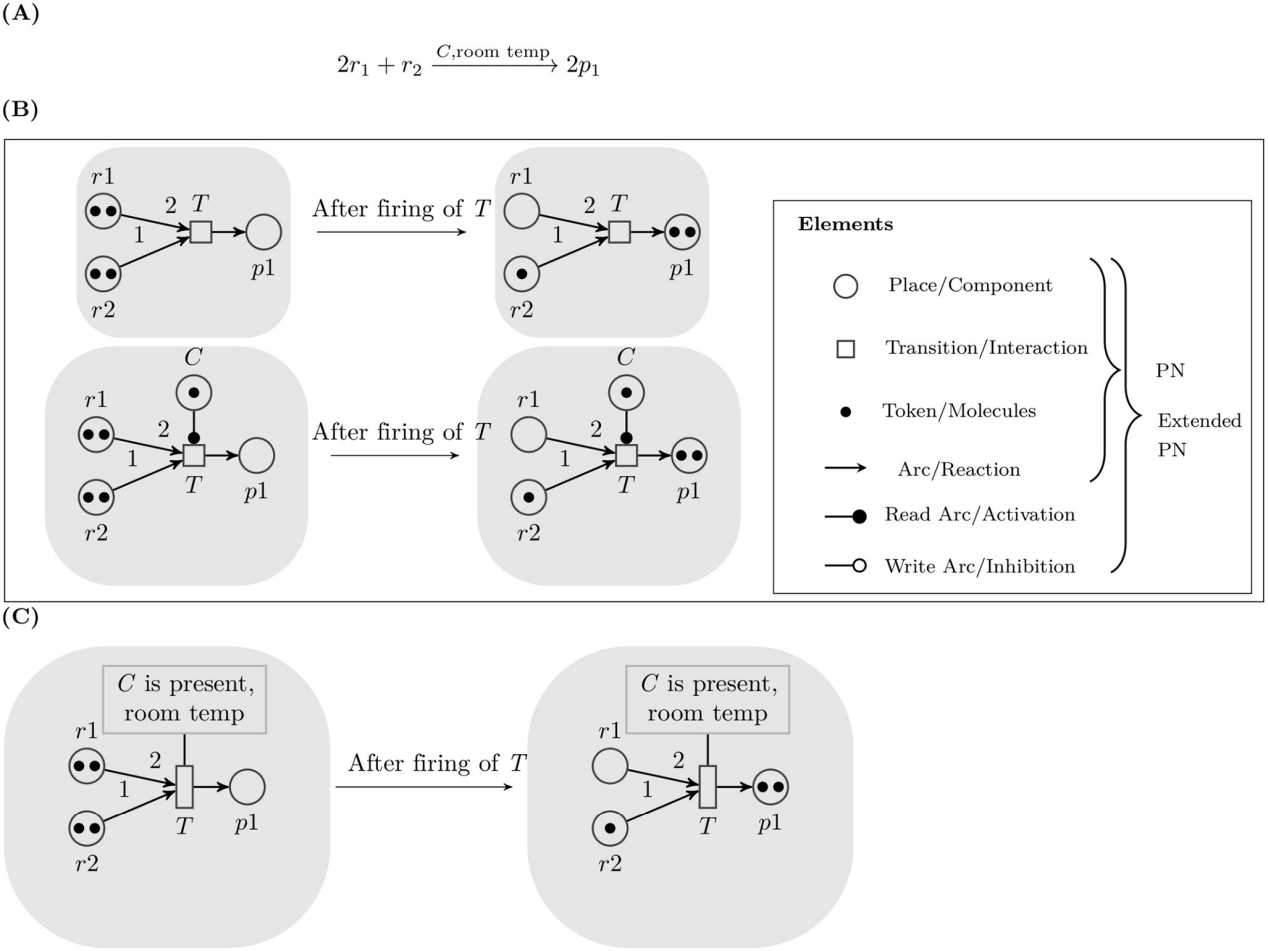
Illustration of Petri net, extended Petri net and LGTS structure representation of a biochemical reaction. (A) A biochemical reaction (B) Petri net and extended Petri net representation of a biochemical reaction. (C) LGTS representation of a biochemical reaction. The box in (C) represents the constraint box for the reaction to happen.

Petri nets have been employed by Durzinsky et al. for the reconstruction of signal transduction as well as gene regulatory networks [31]. They further extended their work and proposed an extended Petri Net model to model biochemical processes that involve catalytic activities along with the consumption and production of components [32, 33]. The extended Petri net structure contains additional *control* nodes (to represent catalysts) that connect to transition nodes using special arcs known as *read arc* (represents activation) and *write arc* (represents inhibition). Fig 1B illustrates the structure of Petri net and extended Petri net for a biochemical reaction given in Fig 1A. A Logic Guarded Transition System (LGTS) [34] is a logic-based transition system proposed as a generalization of Petri nets in the sense that it allows constraint guard on each transition (see Fig 1C). Constraint guards contain constraints that should be fulfilled in order to the transition to fire. The extended Petri net model and the LGTS are both deterministic discrete models. The use of the extended Petri net model and the LGTS system for biochemical reactions are advantageous over other computational models since they facilitate the inclusion of external conditions required for the reactions to happen in the form of *control* nodes and constraint guards. In the LGTS system, constraint guards can also be used for the incorporation of prior knowledge about the system which can help constrain the solution space.

To deal with inherent stochasticity in the biochemical pathways, Vatsa et al. [35] developed a probabilistic extension of the LGTS model, Probabilistic Logic Guarded Transition System (PLGTS), for the network inference of biochemical networks such as signalling networks and metabolic networks. The PLGTS system makes use of the LGTS identifier to identify the transitions of the network and use probabilistic modelling to deal with inherent noise in the data. In PLGTS, each transition node is associated with a probability distribution for its firing. Srinivasan et al. [36] proposed another LGTS based approach for state transition identification in signalling and metabolic networks from noisy data. Though these two probabilistic approaches can work for signalling and metabolic networks, they will not work on gene regulatory networks, since, GRNs differ from signalling and metabolic networks in terms of the components involved as well as their actions.

Biochemical reactions (involved in metabolic and signalling networks) are represented as consumption and production of components, and they happen instantaneously. Therefore, a reaction is modelled using a single transition node in the PLGTS learning approach. In GRNs, however, components are involved in regulatory activities, and no consumption of components takes place. Such regulatory actions can be modelled in the PLGTS, using only control and output places. But unlike signalling and metabolic networks, where a decrease in a protein (or metabolite) concentration represents its consumption, in gene expression data, a decrease in a gene expression level represents gene decay. Moreover, gene expression and gene decay are two separate processes in gene regulation that happen in parallel and thus cannot be modelled using a single transition node in the PLGTS approach. Therefore, the PLGTS approach cannot be used for the inference of GRNs. Considering these specifications, the aim is to develop a Petri net-based approach for GRN inference where gene expression and gene decay are modelled as separate processes.

### Assumptions for GRN inference approach

Depending upon the characteristics of gene regulatory pathways, we have made certain assumptions for the proposed inference approach.

*Assumption 1: Stochasticity*

GRNs are inherently stochastic; that is, there are fluctuations in the process of gene regulation in GRNs. Considering this, the inference approach is designed to be probabilistic in nature to capture the underlying stochasticity from the expression data.

*Assumption 2: First-order Markov process*

In GRN, the regulation of genes takes place in a time series order—when the regulator gene is accumulated in sufficient quantity and activated, then the regulation process initiates, and the gene expression begins to happen. Thus, the regulation of a gene depends on the state of the regulator gene at the previous time point. Thus, the GRN is assumed to be following the first-order Markov process.

*Assumption 3: Discrete model to capture state change*

The gene regulation initiates after the concentration of the regulator gene reaches a threshold, and the regulator gene becomes active [21]. The ON and OFF state of genes at the abstract level can be best monitored in discretized data. The time series discretized data gives us the complete picture of state changes of all gene expression levels. In this work, we have worked our approach with 2-bin (low and high), and 3-bin (low, medium, high) discretized levels of expression data.

*Assumption 4: Restricted number of regulator genes*

In a GRN, a gene can regulate the expression of many target genes but it is unlikely that a large number of regulator genes regulate the expression of a particular target gene. That is, each gene is regulated by a few regulator genes. Thus, our model assumes that each gene has a restricted number of regulators. For this reason, in the case of large gene data sets, appropriate thresholds are used on the number of regulators for each target gene, and a final set of edges is inferred.

### Network inference approach

**Fig 2:**
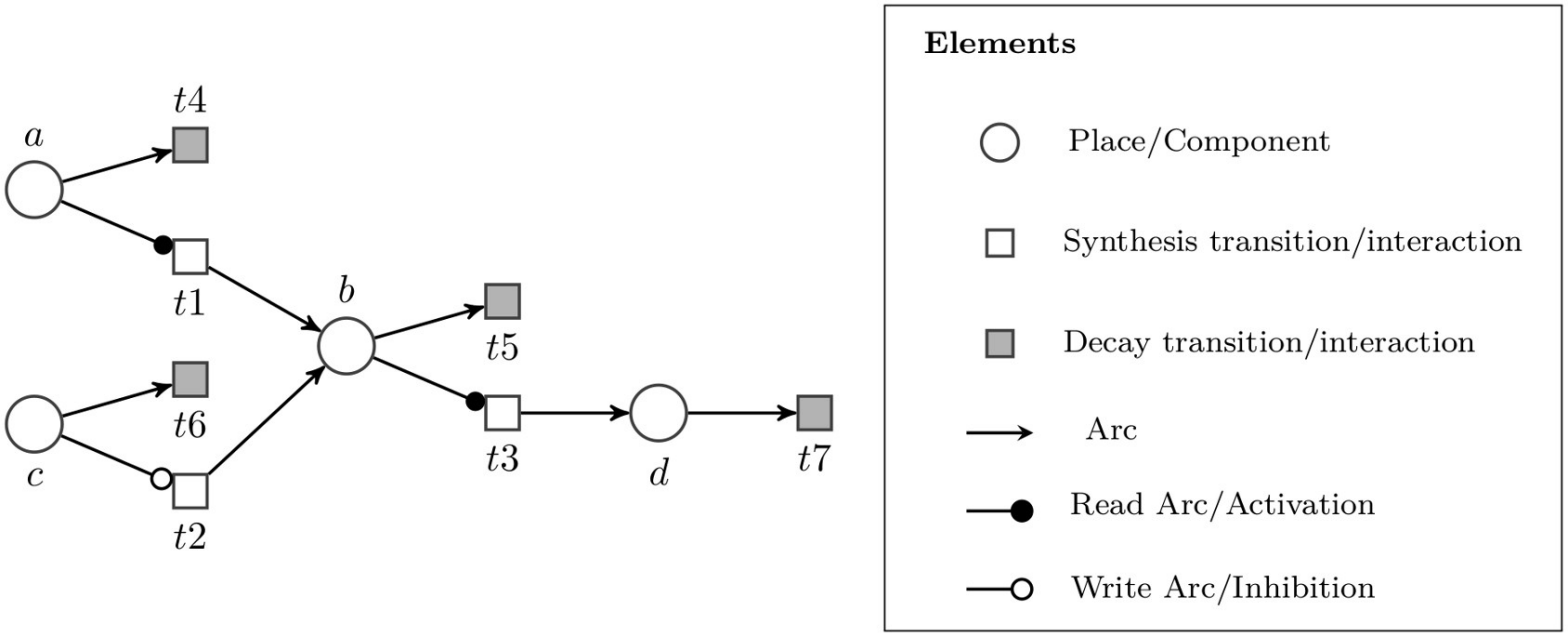
A toy PEPN-GRN structure.

The proposed network inference approach, Probabilistic Extended Petri Net for Gene Regulatory Network (PEPN-GRN) is based on probabilistic extended Petri net model that uses logic rules to represent the underlying mechanism of production and decay of genes in a gene regulation process. It identifies the regulator-target gene pairs by looking at the state transition patterns of gene expression levels in the data. For every adjacent state pair, the approach applies the defined logical rules to identify potential regulator-target gene pairs. Gene decay activity is also captured from the data, and its probability is presented explicitly in the inferred network. Fig 2 illustrates an extended Petri net structure of the PEPN-GRN network. Gene regulation is represented using only the control and output places where a control place represents a regulator gene, and an output place represents a target gene. For gene expression and gene decay, we have two types of transition nodes in the PEPN-GRN representation, namely, *synthesis transition* and *decay transition*, respectively. The synthesis transition is shown as a hollow transition while the decay transition is shown as a grey-colored transition. The regulatory action of activation (or inhibition) between control and output place is shown by read arc (or write arc), respectively.

#### Evidence types and logical rules

In a binary setting, at any pair of adjacent time instants in time series data, a gene can either make a switch or remain constant. Thus, four events are possible:

$$\begin{array}{ccc} \text{Event} & \text{t}-1 & \text{t}\\ \text{Production} & 0 & 1\\ \text{Decay} & 1 & 0\\ \text{Sustained}\;\text{production} & 1 & 1\\ \text{Sustained}\;\text{decay} & 0 & 0 \end{array}$$

A gene is said to be *produced* or *synthesized* when its expression level makes a transition from OFF to ON state (in a binary setting, 0 to 1). While when a gene is getting produced for some time and has attained some high level of expression, we term that instance as *sustained production* of a gene. Similarly for *decay* and *sustained decay* of gene. In production and decay events, gene expression level changes along with two adjacent time points while in sustained production and sustained decay events, gene expression level remains the same. Each such event in a state pair tells us about the action happening on the gene. By observing these states of gene expressions in a time series data, we can see how a particular gene is evolving. Each of the four events of a gene provides evidence about their potential regulator genes. Thus, they serve as four types of evidence to make predictions about the potential regulators of genes. Earlier work on GRN inference using Petri net modelling [32] has considered only the production of the target gene to identify potential regulator genes. In this work, we extend it by using all four evidence types for making predictions about the potential regulator genes. The inference approach uses logical rules defined for four evidence types on the data to infer regulator-target gene pairs. The logical rules are defined for each evidence type as a background knowledge (see Table 1 for logical rules in a binary setting).

**Table 1:** Logical rules for each evidence type for 2-bin discretized data.

**Production evidence**					**Decay evidence**				
Gene	Activation		Inhibition		Gene	Activation		Inhibition	
	*t-1*	*t*	*t-1*	*t*		*t-1*	*t*	*t-1*	*t*
Regulator	1	0 or 1	0	0 or 1	Regulator	0	0 or 1	1	0 or 1
Target	0	1	0	1	Target	1	0	1	0
**Sustained Production evidence**					**Sustained Decay evidence**				
Gene	Activation		Inhibition		Gene	Activation		Inhibition	
	*t-1*	*t*	*t-1*	*t*		*t-1*	*t*	*t-1*	*t*
Regulator	1	0 or 1	0	0 or 1	Regulator	0	0 or 1	1	0 or 1
Target	1	1	1	1	Target	0	0	0	0

Tables show the logical rules used for the four evidence types. In 2-bin discretized data, state values 0 and 1 represent OFF and ON state of a gene, respectively. In each table, the target gene shows its state transition at adjacent time points as per the evidence type. Like in the first table, the state transition of the target gene from OFF (0) to ON (1) represents its production. Each table shows the values a potential regulator gene can take at adjacent time points for both activation and inhibition regulation of the target gene. For instance, for the production of a target gene, a regulator gene (with activation regulation) should be ON (1) at time *t*-1 and can be OFF and ON (0 or 1) at time *t*. For inhibition regulation, it should be OFF (0 value) at time *t*-1 and can be OFF and ON (0 or 1) at time *t*.

We also used 3-bin discretization of gene expression levels where discretized levels 0, 1, and 2 correspond to three states of a gene: low, medium, and high. In a 3-bin setting, a gene is considered to be produced when it makes a transition to a high state, i.e., either from 0 to 2 or from 1 to 2. Similarly, it is considered decayed when it makes a transition to a low state, i.e., either from 2 to 0 or from 1 to 0. Logical rules we consider as providing evidence in 3-bin cases are as given in Table 2.

**Table 2:** Logical rules for each evidence type for 3-bin discretized data.

**Production evidence**					**Decay evidence**				
Gene	Activation		Inhibition		Gene	Activation		Inhibition	
	*t-1*	*t*	*t-1*	*t*		*t-1*	*t*	*t-1*	*t*
Regulator	1/2	0/1/2	0	0/1/2	Regulator	0	0/1/2	1/2	0/1/2
Target	0/1	2	0/1	2	Target	2/1	0	2/1	0
**Sustained Production evidence**					**Sustained Decay evidence**				
Gene	Activation		Inhibition		Gene	Activation		Inhibition	
	*t-1*	*t*	*t-1*	*t*		*t-1*	*t*	*t-1*	*t*
Regulator	1/2	0/1/2	0	0/1/2	Regulator	0	0/1/2	1/2	0/1/2
Target	2	2	2	2	Target	0	0	0	0

Tables show the logical rules used for the four evidence types. In 3-bin discretized data, gene states 0, 1, and 2 represent low, medium, and high state levels, respectively. In each table, the target gene shows its state transition at adjacent time points as per the evidence type. Like in the first table, the state transition of the target gene from low/medium (0/1) to high (2) represents its production. Each table shows the values a potential regulator gene can take at adjacent time points for both activation and inhibition regulation of the target gene. For instance, for the production of a target gene, a regulator gene (with activation regulation) can be medium/high (1/2) at time *t*-1 and can have any level (0, 1 or 2) at time *t*. For inhibition regulation, it should be at low level (0) at time *t*-1 and can have any level (0, 1 or 2) at time *t*.

#### PEPN-GRN framework

Consider a time series expression data set *D* that contains the expression values of *p* genes measured at *n* time points.

$$\begin{array}{c} D=\{X^{t_{1}},X^{t_{2}},\cdots ,X^{t_{n}}\} \end{array}$$ (1)

Each column vector $X^{t_{l}}$ represents expression values of *p* genes at *t*_*l*_ time point where *l* = 1, 2, …, *n*.

$$\begin{array}{c} X^{t_{l}}={\left[\text{expr}{\left(x_{1}\right)}^{t_{l}},\text{expr}{\left(x_{2}\right)}^{t_{l}},\cdots ,\text{expr}{\left(x_{p}\right)}^{t_{l}}\right]}^{T} \end{array}$$ (2)

It basically represents the state of the network at time point *t*_*l*_. For every adjacent state pair i.e., (*X*^*t*−1^, *X*^*t*^) for *t* = 2, 3, …, *n*, using logical rules, the method retrieves the edges in the form of (*regulator, target, sign*) triplets.

$$\begin{array}{c} {\text{Edges}}_{e}\left(X^{t-1},X^{t}\right)=\left\{\left(x_{i},x_{j},\text{sign}\right)\right\} \end{array}$$ (3)

where *i*, *j* ∈ {1, 2, …, *p*}, *sign* = {1, 0}, for each evidence *e* ∈ {*e*1, *e*2, *e*3, *e*4}.

Here *x*_*i*_ and *x*_*j*_ represents regulator gene and target gene respectively. Edge sign 1 and 0 represents activatory and inhibitory edge respectively.

Let *StatePairs*(*D*) denotes all adjacent state pairs in the data set D,

$$\begin{array}{c} \text{StatePairs}\left(D\right)=\{\left(X^{t_{1}},X^{t_{2}}\right),\left(X^{t_{2}},X^{t_{3}}\right),\cdots ,\left(X^{t_{n-1}},X^{t_{n}}\right)\} \end{array}$$ (4)

Then, the edge set *Edges* retrieved from all adjacent state pairs for evidence *e* is:

$$\begin{array}{c} {\text{Edges}}_{e}\left(\text{StatePairs}\left(D\right)\right)=\cup_{\left(X^{t-1},X^{t}\right)\in \text{StatePairs}\left(D\right)}{\text{Edges}}_{e}\left(X^{t-1},X^{t}\right) \end{array}$$ (5)

The edge set corresponding to each evidence type may contain many regulators with both regulation signs for each target gene. Thus, the probability of each regulator-sign pair (*x*_*i*_, *sign*) is computed for each target gene *x*_*j*_ as:

$$\begin{array}{c} {\text{Pr}}_{e}\left(x_{i},x_{j},\text{sign}\right)=\frac{C_{e}\left(x_{i},x_{j},\text{sign}\right)}{C_{e}\left(x_{j}\right)} \end{array}$$ (6)

where the numerator represents the count of the edge (*x*_*i*_, *x*_*j*_, *sign*) in the edge set for evidence *e* and denominator represents the count or the number of times the target gene *x*_*j*_ was present in evidence *e*. For instance, for decay evidence *DE*, *C*_*DE*_(*x*_*j*_) represents count of the number of times the target gene *x*_*j*_ makes the transition 1 → 0 in all state pairs in data D.

The final score for each edge is computed by taking the average of all evidence type probabilities:

$$\begin{array}{c} \text{Score}\left(x_{i},x_{j},\text{sign}\right)=\frac{\sum_{e=1}^{4}{\text{Pr}}_{e}\left(x_{i},x_{j},\text{sign}\right)}{4} \end{array}$$ (7)

In cases where the triplet (*x*_*i*_, *x*_*j*_, *sign*) is present in the edge set corresponding to evidence let us say *e1* but absent in evidence *e2*, the probability of the triplet for evidence *e2* is taken to be 0 while computing the score. Thus for any gene pair even if any of the evidence types is missing from the data, still all evidence types are considered while computing the final score for the edge.

The edge set returned after this step contains edges with their scores. In the final step of the PEPN-GRN approach, regulation sign selection is made for all edges. For edges with different scores across two signs, the higher score triplet is selected. In a GRN, any regulator gene can either positively regulate the target gene or negatively regulate it but cannot have both regulations. Therefore, the edges with the same score across two signs are discarded, as this signifies a clear case of a false positive. The final edge set contains the edges with a single regulation sign. The score of each edge represents its significance, the higher the score, the more significant is the edge. It should be noted that, here the score of an edge does not represent the probability with which the edge should be present in the network but represents the probability of the edge containing a particular regulation sign assuming that the edge is present. Thus, the score of an edge across two regulation signs sums up to be 1.

The decay probability of each gene *x*_*i*_ is computed as:

$$\begin{array}{c} \text{DecayProb}\left(x_{i}\right)=\frac{C_{\text{decay}}\left(x_{i}\right)}{C_{\text{occ}}\left(x_{i}\right)} \end{array}$$ (8)

where the numerator represents the decay count of the gene *x*_*i*_ and the denominator represents the occurrence count of the gene *x*_*i*_. If the Difference matrix *Diff* of the time series data *D* is defined as:

$$\begin{array}{c} \text{Diff}=\cup_{\left(X^{t-1},X^{t}\right)\in \text{StatePairs}\left(D\right)}\left(X^{t}-X^{t-1}\right) \end{array}$$ (9)

Each *i*^*th*^ row in the difference matrix, *Diff*(*i*,:) shows the difference of expression values of *x*_*i*_ gene at adjacent time points. Therefore,

$$\begin{array}{c} \begin{array}{cc} C_{\text{decay}}\left(x_{i}\right) & =\text{sum}(\text{Diff}(i,:)<0)\;\text{and}\;\\ C_{\text{occ}}\left(x_{i}\right) & =\text{sum}(D(i,:)>0) \end{array} \end{array}$$ (10)

The estimated decay probability of each gene shows how often the gene decays in the given data set.

**Fig 3:**
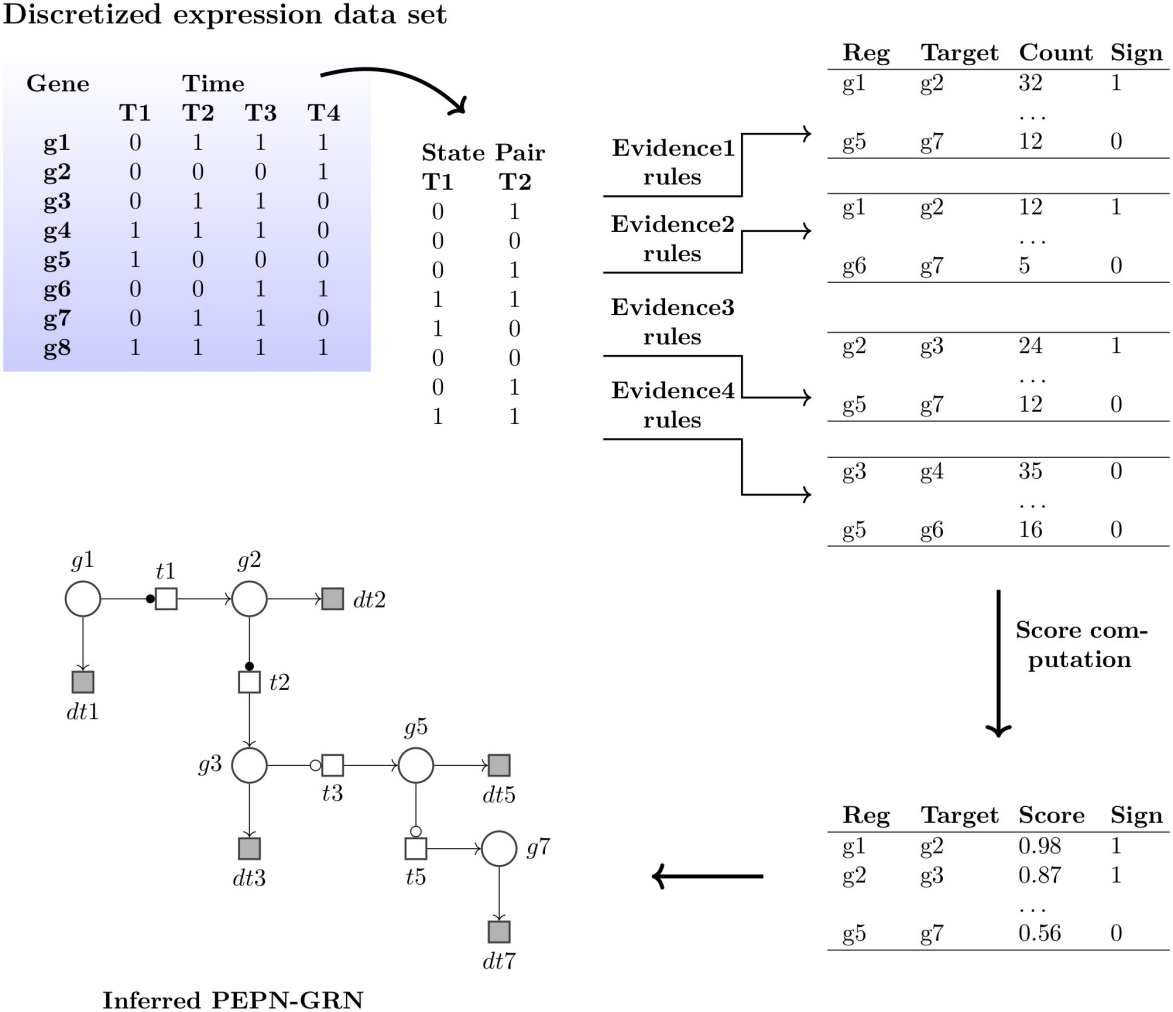
Work flow of the PEPN-GRN method. The main steps of the PEPN-GRN approach are shown here. For each state pair taken at adjacent time points from the discretized data, logical rules of evidence types are applied that return a set of edges with their evidence counts. Evidence probabilities are computed using these evidence counts. Next, score computation is done using different aggregation methods. Finally using a suitable threshold on the edge scores, a PEPN-GRN network is inferred.

Fig 3 presents the work flow of the PEPN-GRN approach.

#### Weighted aggregation of probabilities

We now introduce three variants of the PEPN-GRN approach on the basis of weight aggregation of evidence probabilities to compute the final score of edges.

*PEPN-GRN_v1*. In the first variant, the final score of an edge is computed by taking the average of all evidence probabilities. No other weights are used in the score computation. This is termed as an unweighted aggregation basis of probabilities.

*PEPN-GRN_v2*. In the second variant, instead of individual evidence probabilities, we use raw counts of evidence types for score computation. This variant is termed as a weighted aggregation of probabilities. Aggregation is done as:

$$\begin{array}{c} \text{Score}\left(x_{i},x_{j},\text{sign}\right)=\frac{\sum_{e=1}^{4}C_{e}\left(x_{i},x_{j},\text{sign}\right)}{\sum_{e=1}^{4}C_{e}\left(x_{j}\right)} \end{array}$$ (11)

Where the numerator refers to the sum of counts of an edge in each evidence *e* and the denominator refers to the sum of counts of the target gene in each evidence. This way of aggregation allows some kind of weights on the evidence probabilities though we are not using any weights specifically here. The weighting is in accordance with the occurrence frequency of each evidence. Evidence that occurs less often will contribute less in the overall score, while high-frequency evidence will contribute more.

*PEPN-GRN_v3*. The above two variants assumed that all evidence types contribute equally to the activation and inhibition of a gene. However, there is a possibility that some evidence types are more informative about gene activation or inhibition than other evidence types. Using this motivation, we propose the third variant, a supervised learning approach of the PEPN-GRN method that assigns weight to each evidence type as per their usefulness for predicting an inferred activation or inhibition interaction. The PEPN-GRN_v3 first identifies the individual evidence probabilities and then employs a supervised learning approach to learn the weights for each evidence type. After identifying the weights, a final edge probability (score) is computed using the weights and the individual evidence probabilities. The score computed for each edge represents the probability of an edge being present in the network.

We used the Logistic Regression (LR) model to learn the evidence weights separately for activation as well as inhibition edges. The model uses the logistic function to predict the value of probability between 0 and 1. The logistic function is defined as follows:

$$\begin{array}{c} \begin{array}{cc} \sigma (z) & =\frac{1}{1+e^{-z}}\\ \;\text{where}\;z & =w_{0}+\sum_{i=1}^{n}\left(w_{i}*x_{i}\right) \end{array} \end{array}$$ (12)

where *x*_*i*_ and *w*_*i*_ represents the *i*^*th*^ feature and coefficient for *i*^*th*^ feature respectively for *i* = 1, …, *n* and *n* is the total number of features. *w*_0_ represents the bias. *σ*(*z*) represents the predicted probability of an example to lie in class 1. This probability is computed for all *m* examples.

In our case, *m* is the total number of possible edges, the *x*_*i*_ represent the different evidence type probabilities, and the *w*_*i*_ represent their coefficients/weights. Here, since we have four types of evidence, *i* ranges from 1 to 4.

The logistic regression model is applied to learn the weight *w*_*i*_ for each evidence type on the training edge set. The weights are learned using the evidence probabilities of edges (features) and their class labels (edge present or absent). Thus, if the training set consists of *m* edges (examples), the class label would be a vector *Y*^*T*^ of size *m* × 1, and the feature matrix would be a matrix *X* of size *m* × 4. The class label vector contains entries either “0” or “1” that represent the absence or the presence of edges, respectively. Upon training, the logistic regression model returns a vector *W* = [*w*_0_, *w*_1_, …, *w*_4_]. The trained model is applied to the feature matrix *X’* of the test edges to predict the probability of each edge having class label 1. A probability threshold of 0.5 is used to classify the test edges into the two classes. Examples with predicted probability *P* > 0.5 are classified as class 1 and others as class 0.

For the ideal performance of the LR model, the class distribution of the examples in the training set should be *balanced*; that is, the number of positive and negative examples should be similar. However, in real scenarios like GRN inference, often, the case of imbalanced data sets is found. In a gene regulatory network, a single gene can regulate many other genes, but it is unlikely that a gene is regulated by many other regulator genes at the same time. Therefore, in a GRN of a large number of genes, only a few regulator genes (TFs) regulate the target genes. Thus the number of positive examples (actual interactions) is less in comparison to the number of negative examples (non-existent interactions). In the training data, if the class distribution is too skewed, and the negative examples are over-represented, the learned weights will tend to be biased towards the negative examples, and the prediction accuracy would decrease. Therefore, we have used the Synthetic Minority Oversampling Technique (SMOTE) resampling technique to make the data set balanced. Synthetic Minority Oversampling TEchnique (SMOTE) [37] is an upsampling technique where new examples from the minority class are generated synthetically.

The pseudo code for the PEPN-GRN_v3 approach is given as Algorithm 1. The algorithm uses the function *edge_evidence_probability* which is given as Algorithm 2. Functions of different evidence types in Algorithm 2 use logical rules given in Tables 1 and 2.

**Algorithm 1**: PEPN-GRN_v3

**Data**: Time series data set $D=\{X^{t_{1}},X^{t_{2}},\ldots ,X^{t_{n}}\}$; ⊳ where each column vector $X^{t_{l}}$ represents expression values of *p* genes at *t*_*l*_ time point where *l* = 1, 2, …, *n*.

**Result**: Ranked edges of form (*r,t,s,score*); ⊳ where *r* is regulator gene, *t* is target gene, *s* is regulation sign, and *score* is significance score.

1 {*R*, *T*, *S*, *Pr*} = function *edge_evidence_prob* (*D*)

2 Let Edges *E* = {*R*, *T*, *S*}; ⊳ where *R* is column vector of regulator genes, *T* is column vector of target genes, and *S* is a column vector of regulation signs for corresponding gene pair in (*R*, *T*)

3 Let Edge_features *F* = $\left\{{\text{Pr}}_{e_{1}}\left(E\right),{\text{Pr}}_{e_{2}}\left(E\right),{\text{Pr}}_{e_{3}}\left(E\right),{\text{Pr}}_{e_{4}}\left(E\right)\right\}$; ⊳ where each column vector ${\text{Pr}}_{e_{l}}\left(E\right)$ represents *l*^*th*^ evidence probabilities of edges in *E*.

4 Edge_labels *L* = [*L*_1_, *L*_2_, …, *L*_*p*_]′; ⊳ a column vector of *c* class labels for *p* edges.

5 *E* = (*E*_1_∪*E*_2_∪…∪*E*_*k*_); ⊳ for *k-fold* validation, divide *E* into *k* equal folds

6 **for** *fold = 1 To k* **do**

7  *Ts_edges* = *E*_*fold*_

8  *Tr_edges* = *E*\*E*_*fold*_

9  *Ts_features* = F for edges ∈ *Ts_edges*; ⊳ For activation edges

10  *Tr_edges_act* = *Tr_edges* with sign *s* 1, *Tr_features_act* = *F* for edges ∈ *Tr_edges_act*, *Tr_labels_act* = *L* for edges ∈ *Tr_edges_act*

11  {*Tr*_*f*, *Tr*_*l*} = *smote*(*Tr_features_act, Tr_labels_act*); ⊳ *Tr_f* and *Tr_l* are smote upsampled features and labels

12  *wt* = *logistic_regression*(*Tr_f, Tr_l*); ⊳ *wt*are weights learned for features using logistic regression

13  *Ts_score_act* = *model_fit*(*wt, Ts_features*); ⊳ *Ts_score_act* is a column vector test accuracy score when model is fit over test features

14  Repeat steps 10–13 for inhibition edges

15  *Ts_score* = *max*(*Ts_score_act*, *Ts_score_inh*)

16  Final_edges = [*Ts_edges*,*Ts_score*]

17 Return Final_edges

**Algorithm 2**: function (*R*,*T*,*S*,*Pr*) = *edge_evidence_prob*(*D*)

**Data**: Time series data set $D=\{X^{t_{1}},X^{t_{2}},\ldots ,X^{t_{n}}\}$

Predefined regulator genes *regulators*

**Result**: Edges of form (*R,T,S,Pr*); ⊳ where *R* is a column vector of regulator genes, *T* is a column vector of target genes, *S* is a column vector of regulation sign, and *Pr* is a matrix of evidence probabilities for each edge.

1 $S=\{\left(X^{t_{1}},X^{t_{2}}\right),\left(X^{t_{2}},X^{t_{3}}\right),\ldots ,\left(X^{t_{n-1}},X^{t_{n}}\right)\}$; ⊳ *S* is a set of adjacent state pairs

2 **for** *each state pair s*_*i*_ ∈ *S where* 1 ≤ *i* ≤ |*S*|

3 **do**

4  Set *prod_edges* = ∅, *decay_edges* = ∅, *sus_prod_edges* = ∅, *sus_decay_edges* = ∅

5  Difference vector $d=X^{t_{i+1}}-X^{t_{i}}$

6  Find gene indexes *prod_idx* where *d* > 0

7  **for** *each idx* ∈ *prod_idx* **do**

8   (*reg, sign*) = function *prod_evidence*($X^{t_{i}}$, *idx*, *regulators*)

9   Update *prod_edges* = *prod_edges* ∪ (*reg,tar,sign*); ⊳ where *tar* is the gene at *idx*

10  end

11  Find gene indexes *decay_idx* where *d* < 0

12  **for** *each idx* ∈ *decay_idx* **do**

13   (*reg, sign*) = function *decay_evidence*($X^{t_{i}}$, *idx*, *regulators*)

14   Update decay_edges = decay_edges ∪ (*reg,tar,sign*); ⊳ where *tar* is the gene at *idx*

15  end

16  Find rest gene indexes *rest_idx* = *all_idx* \ (*decay_idx* ∪ *prod_idx*)

17  **for** each *idx* ∈ *rest_idx*

18   **do**

19   *gene_pre* = $X^{t_{i}}\left(idx\right)$; ⊳ gene expression at pre state

20   *gene_post* = $X^{t_{i+1}}\left(idx\right)$; ⊳ gene expression at post state

21   **if** *gene_pre* == 0 and *gene_post* == 0 **then**

22    *(reg, sign)* = function *sus_decay_evidence*($X^{t_{i}}$, *idx*, *regulators*)

23    Update *sus_decay_edges* = *sus_decay_edges*∪(*reg, tar, sign*); ⊳ where *tar* is the gene at *idx*

24   **else if** *gene_pre* == 1 and *gene_post* == 1 **then**

25    *(reg, sign)* = function *sus_prod_evidence*($X^{t_{i}}$, *idx*, *regulators*)

26    Update *sus_prod_edges* = *sus_prod_edges* ∪ (*reg,tar,sign*); ⊳ where *tar* is the gene at *idx*

27  end

28 end

29 Set *edges* = ∅

30 **for** each evidence *e*_*i*_ where *i* = {1, 2, 3, 4} **do**

31  **for** each target gene *x*_*j*_ where *j* = 1: *p* **do**

32   Compute edge probability ${\text{Pr}}_{e_{i}}\left(\text{reg},x_{j},\text{sign}\right)=\frac{C_{e_{i}}\left(\text{reg},x_{j},\text{sign}\right)}{C_{e_{i}}\left(x_{j}\right)}$; ⊳ where numerator represents the count of edge in evidence *e*_*i*_ and denominator represents the count of *x*_*j*_ in evidence *e*_*i*_.

33   Update *edges* = *edges* ∪ (*reg*,*x*_*j*_,*sign*,${\text{Pr}}_{e_{i}}$)

34  end

35 end

### Discretization methods

Yong Li et al. [38] discussed different discretization methods that can be used for gene expression data. In this work, we have used three discretization methods namely, Equal Width Discretization (EWD), Equal Frequency Discretization (EFD), and the K-means method since they discretize the data along each gene level. The methods discretize each gene’s expression values into *k* intervals, where 1 < *k* < *n* where n is the number of time points in the data.

Define the data matrix *D* as a *p* × *n* matrix where *p* is the number of genes and *n* is the number of time points in all time series. *D*(*p*,:) denotes the expression levels of *p*^*th*^ gene at all the time points in all time series. Let *k* be the number of intervals into which we want to discretize our data. Setting the value of *k* to 2 leads to binary discretization, setting it to 3 leads to ternary discretization and so on. The number of intervals is a user-defined parameter.

EWD method [39–41] discretizes the expression levels of each gene into *k* intervals such that each interval has the same width. The width threshold is computed as

$$\begin{array}{c} \text{Thresh}=\frac{\left(\text{max}(D(p,:))-\text{min}(D(p,:))\right)}{k} \end{array}$$ (13)

For any *k* ranging from *2* to *l* where *l* < *n*,

$$\begin{array}{c} \text{interval}\;\text{1}\;\text{threshold}=\text{min}(D(p,:))+\text{Thresh}\\ \text{interval}\;\text{2}\;\text{threshold}=\text{interval}\;\text{1}+\text{Thresh}\\ \vdots \\ \text{interval}\;\text{k}\;\text{threshold}=\text{interval}\;\text{k-1}+\text{Thresh} \end{array}$$

EFD method [39–41] discretizes the data matrix *D* by first sorting each gene row *D*(*p*,:) and then dividing it into *k* intervals such that each interval contains expression levels with the same frequency.

K-means method [42] discretizes the data matrix *D* by applying K-means clustering on each gene row *D*(*p*,:) such that the neighboring expression levels are combined in the same cluster.

### Assessment

For performance assessment of inference models, two evaluation metrics are used: *Receiver Operating Characteristic (ROC)* curve and *Precision-Recall (PR)* curve. The ROC curve shows the plot between the fraction of correctly classified positive examples over the total positive examples (known as True Positive Rate (TPR)) and the fraction of incorrectly classified negative examples over the total negative examples (known as False Positive Rate (FPR)). With TPR on the y-axis and FPR on the x-axis, the area under the ROC curve (AUROC) can be regarded as a metric of the performance of a model. It basically represents how well the model distinguishes between the two classes.

The Precision-Recall curve plots the Precision and Recall values obtained for different thresholds. The *Precision* (also known as Positive Predictive Value (PPV)) is defined as the fraction of correctly classified positive examples among the total number of examples classified as positive. The *Recall* or *Sensitivity* is the same as TPR. With Precision on the y-axis and Recall at the x-axis, the area under the PR curve (AUPR) defines the performance of a model.

For the experiments on the DREAM4 challenge data sets, we have used the scoring function provided by the DREAM organizers in the DREAMTools package [43]. The scoring function returns AUROC and AUPR scores that are computed as the average of the log of p-values of AUROC and AUPR of five networks respectively. AUROC score and AUPR score represents the single overall score of AUROC and AUPR obtained over *n* networks. The AUROC and AUPR scores are computed as:

$$\begin{array}{c} {\text{Score}}_{\text{AUROC}}=-\frac{1}{n}\sum_{i=1}^{n=5}\left({\text{log}}_{10}\left(p_{\text{AUROC},i}\right)\right) \end{array}$$ (14)

$$\begin{array}{c} {\text{Score}}_{\text{AUPR}}=-\frac{1}{n}\sum_{i=1}^{n=5}\left({\text{log}}_{10}\left(p_{\text{AUPR},i}\right)\right) \end{array}$$ (15)

The final score is the mean of AUROC score and AUPR score. It denotes the overall performance of the inference method.

## Results

We first evaluated the performance of the PEPN-GRN variant methods and other inference methods on the DREAM4 *in silico* network challenge data sets. We then perform the evaluation on real expression data set of *E. coli* taken from the DREAM5 Challenge.

Other network inference methods used for performance evaluation on the DREAM4 data set are Bayesian-based scanBMA [44], regression-based DBN inference method G1DBN [45], Random Forest-based dynGENIE3 [17], and mutual information-based ARACNE, CLR, and MRNET [46]. Specifically, we have used the G1DBN method from the *G1DBN* package [47], scanBMA from *networkBMA* package [48], mutual information-based methods from the *minet* package [49], and an R implementation of dynGENIE3 [50]. Note that we have not taken the metrics AUROC and AUPR from other papers but re-run these methods on our own to obtain the score matrix using each inference method. We have then used the DREAMTools package to assess the inferred networks. For details on the application of these inference methods, please refer to data in S1 Appendix.

### DREAM4 *in silico* network challenge data set

We first compared the performance of PEPN-GRN variant methods and other inference methods on discretized DREAM4 data sets. Data sets are discretized into 2 bins (binary) and 3 bins (ternary) using three chosen discretization methods, namely, EFD, EWD, and K-means. Second, to evaluate the impact of discretization, we compared the performance of inference methods (other than PEPN-GRN variant methods) on continuous and discretized DREAM4 data sets.

Here, in the PEPN-GRN_v3 variant, k-fold cross-validation method is used with *k* = 5 to divide the data sets into the train and test sets. For each fold *f*, in 5-fold cross-validation where *f* = 1, 2, …, 5, the *f*^*th*^ network is taken as the test network and the rest of the networks are considered as train networks. First, for each network in the train and test sets, evidence type probabilities are computed for each edge in the network. In 10-gene data sets, each network contains a total of 90 edges (excluding the self edges). When both regulatory signs (+ and -) are considered, total possible edges become 180, 90 activation edges and 90 inhibitory edges. In any fold in 5-fold cross-validation, the train set consists of four networks. Therefore, the feature matrix (made of evidence probabilities) contains 180*4 = 720 rows. Each row corresponds to the features of an edge. The feature matrix of the test set contains 180 rows. Since the role of each evidence type may differ for the activatory and inhibitory regulations, two logistic regression models are learned for the two sets of edges: activatory and inhibitory.

The performance of the three variants of the PEPN-GRN approach is compared on the DREAM4 data sets using average AUROC and average AUPR metrics (averaged over five networks) (see Table 3). Note that here, we have used scores of all gene pairs to compute the AUROC and AUPR metrics, and no thresholding is done for the selection of the final set of gene pairs since the number of genes is less in the DREAM4 *in silico* networks.

**Table 3:** Performance of the three variants of the PEPN-GRN approach on EFD, EWD, K-means discretized DREAM4 data sets. Average baseline AUPRs for 10-gene and 100-gene data sets are 0.158 and 0.02 respectively.

Average AUROC / AUPR						
	**DREAM4 10-gene**			**DREAM4 100-gene**		
**Method**	**EFD 2-bin**	**EWD 2-bin**	**K-means 2-bin**	**EFD 2-bin**	**EWD 2-bin**	**K-means 2-bin**
PEPN-GRN_v1	0.612 / 0.376	0.694 / 0.348	0.656 / 0.368	0.656 / 0.1	0.64 / 0.052	0.638 / 0.066
PEPN-GRN_v2	0.632 / 0.39	0.59 / 0.298	0.594 / 0.322	0.674 / 0.094	0.576 / 0.032	0.598 / 0.05
PEPN-GRN_v3	0.594 / 0.338	0.706 / 0.354	**0.678 / 0.39**	**0.702 / 0.114**	0.644 / 0.062	0.67 / 0.084
	**DREAM4 10-gene**			**DREAM4 100-gene**		
**Method**	**EFD 3-bin**	**EWD 3-bin**	**K-means 3-bin**	**EFD 3-bin**	**EWD 3-bin**	**K-means 3-bin**
PEPN-GRN_v1	0.698 / 0.404	0.714 / 0.41	0.704 / 0.412	0.696 / 0.112	0.668 / 0.088	0.672 / 0.098
PEPN-GRN_v2	0.704 / 0.404	0.594 / 0.37	0.582 / 0.318	0.696 / 0.108	0.588 / 0.068	0.622 / 0.082
PEPN-GRN_v3	**0.71 / 0.4**	0.704 / 0.358	0.7 / 0.354	**0.728 / 0.132**	0.688 / 0.08	0.698 / 0.1

The performance comparison of the PEPN-GRN variants on 2-bin and 3-bin discretized data sets shows an improvement on 3-bin as compared to 2-bin. The average AUROC increases by 6–11%, and the average AUPR increases by 3–6%. Comparing the performances of the PEPN-GRN variants in the 2-bin case, PEPN-GRN_v3 obtained the best performance with an average AUROC of 0.678 and AUPR 0.39 on 10-gene K-means data set while obtaining an average AUROC of 0.702 and AUPR of 0.114 on 100-gene EFD data set. In the 3-bin case, PEPN-GRN_v3 obtained average AUROC and AUPR of 0.71 and 0.4, respectively in the 10-gene EFD data set. Almost similar performance is obtained by the other two variants. However, on 100-gene data sets, PEPN-GRN_v3 performs the best with AUROC of 0.728 and AUPR of 0.132 on the EFD data set. Thus, on small data sets (like 10-gene), the PEPN-GRN_v3 gives almost a similar performance as the other two variants, while as the data set size increases (100-gene), the performance becomes much improved. The improvement is likely as the performance of a supervised learning model improves with the increase in data. Among the three discretization methods used, it is seen that the PEPN-GRN variants give the best results on EFD data sets and perform worst on EWD data sets.

**Fig 4:**
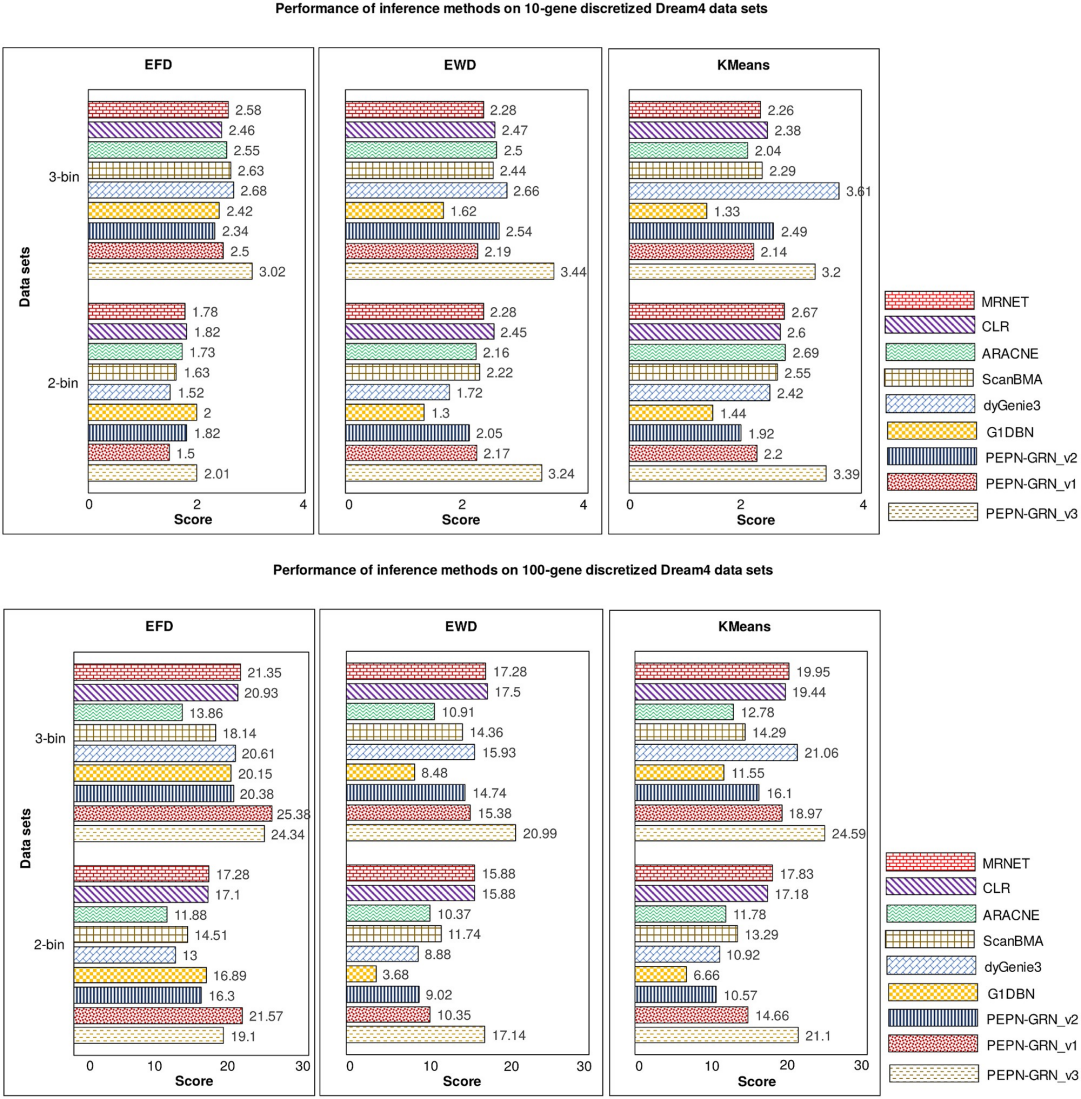
Performance evaluation of inference methods on discretized 10-gene and 100-gene DREAM4 data sets. For different discretization methods, EFD, EWD, and K-means, the prediction performance of directed unsigned networks is shown as scores for 2-bin and 3-bin discretized 10-gene and 100-gene DREAM4 networks. The performance of all inference methods on 3-bin discretized data sets outperformed the performance on 2-bin discretized data sets. On 10-gene data sets, the best performing methods are G1DBN (in case of 2-bin discretization) with a score of 3.39, and dynGenie3 (in case of 3-bin discretization) with an overall score of 3.61. On 100-gene data sets, the best performing method is PEPN-GRN_v3 in both 2-bin and 3-bin discretization with a score of 21.57 and 25.38, respectively.

Comparative analysis of the performance of the PEPN-GRN variants and other inference methods on discretized DREAM4 data sets is shown in Fig 4. The bar plots show that all inference methods performed better on 3-bin discretized data than on 2-bin data. On 10-gene data sets, in the case of 2-bin discretization, the best performing method is the G1DBN with a score of 3.39, while in the case of 3-bin discretization, the best performing method is dynGenie3 with an overall score of 3.61. Both methods obtained their best performance on K-means discretized data sets. On 100-gene data sets, the best performance is obtained by PEPN-GRN_v3 in both 2-bin and 3-bin discretization with a score of 21.57 and 25.38, respectively. Both the scores are obtained on EFD discretized data sets. On EWD and K-means data sets, the best performance is achieved by G1DBN in both 2-bin and 3-bin cases. Thus, on 10-gene data sets, two top-performing methods are G1DBN and dynGenie3, while on 100-gene data sets, two top-performing methods are G1DBN and PEPN-GRN_v3.

Examining the performance of PEPN-GRN_v3, in the EFD 2-bin 10-gene data case, the performance of the PEPN-GRN_v3 is even lower than the other two PEPN-GRN variants; however, in the EFD 3-bin 10-gene data case, the performance is better than the two variants. This suggests that increasing the number of bins also helps in the predictions in the PEPN-GRN_v3 approach. The reason for relatively poor performance on 10-gene data as compared to the 100-gene data is that the model could not train well with fewer data. A sufficiently large amount of training data is crucial for supervised learning approaches. Thus the PEPN-GRN_v3 method is expected to perform better when trained on the large labeled data sets.

From the 100-gene results, the best discretizing method for the G1DBN and the dynGenie3 is K-means (though their performance on EFD is also quite close) and for PEPN-GRN variants, it is EFD. Other inference methods such as ScanBMA, and mutual information-based methods also performed their best on the EFD discretized data. It was observed that all the methods have the least performance on the EWD data in the 100-gene case. Thus, EFD and K-means proved to be good discretization methods.

In the PEPN-GRN_v3 approach, final scores obtained for the edges are their probabilities of being present in the network. The likelihood assessment of inferred edges in learned networks reflects the probabilistic aspect of the approach. Likelihood assessment of the learned network with respect to the ground truth network is done on 2-bin and 3-bin EFD discretized 100-gene DREAM4 networks (see Table 4). Between 2-bin and 3-bin data sets, for most networks, likelihood is lower in the case of 2-bin data sets which signifies better edge prediction on 3-bin data sets as compared to 2-bin data. On network 3, the likelihood of ground truth network is lower for 2-bin data but the likelihood of edges present in ground truth network is lower for 3-bin data which shows that edges present in the ground truth network are predicted with higher accuracy in 2-bin data for this particular network. Such kind of assessment cannot be done on other competing methods where inferred edges are ranked using some different kinds of scores. For instance, the G1DBN approach ranks the edges based on their p-values which are not exactly probabilities.

**Table 4:** Likelihood assessment of inferred edges on EFD discretized 100-gene DREAM4 networks using the PEPN-GRN_v3 approach.

	Likelihood of ground truth network		Likelihood of only edges present in ground truth network	
	2-bin data	3-bin data	2-bin data	3-bin data
Net 1	6.24e-33	3.78e-33	8.81e-34	4.43e-30
Net 2	1.002e-34	1.33e-34	3.56e-50	6.21e-51
Net 3	2.18e-35	2.45e-32	1.73e-37	2.42e-43
Net 4	8.98e-32	1.079e-32	1.76e-48	2.54e-43
Net 5	8.75e-36	8.24e-33	6.82e-42	1.75e-40

#### Impact of discretization

Data discretization leads to information loss to some extent and hence, likely impacts the performance of the inference technique. To estimate the impact, we have compared the performance of inference methods (other than the PEPN-GRN variant methods) on continuous data and discretized data. We first analyzed the results on the continuous DREAM4 data sets, as shown in Table 5. According to the overall score metric achieved by all methods, the G1DBN method performs best on 10-gene data, where it obtained an overall score of 3.79 while the dynGenie3 approach obtained a slightly lower score of 3.68. On 100-gene data, the dynGenie3 method achieved the highest overall score of 37.96, while the G1DBN scores a much lower score of 27.8. Thus on the continuous data, the best performing inference technique is dynGenie3.

**Table 5:** Performance evaluation of inference methods on DREAM4 10-gene and 100-gene continuous data sets.

	cont. DREAM4 data	
	10-gene	100-gene
Method	Score	Score
G1DBN	**3.79**	27.8
dynGenie3	3.68	**37.96**
scanBMA	3.22	18.64
ARACNE	2.195	15.397
CLR	2.12	22.9
MRNET	2.489	23.04

The performance is evaluated using the overall score metric computed on all five data sets of 10-gene and 100-gene networks. Numbers in bold represent the highest score achieved.

**Fig 5:**
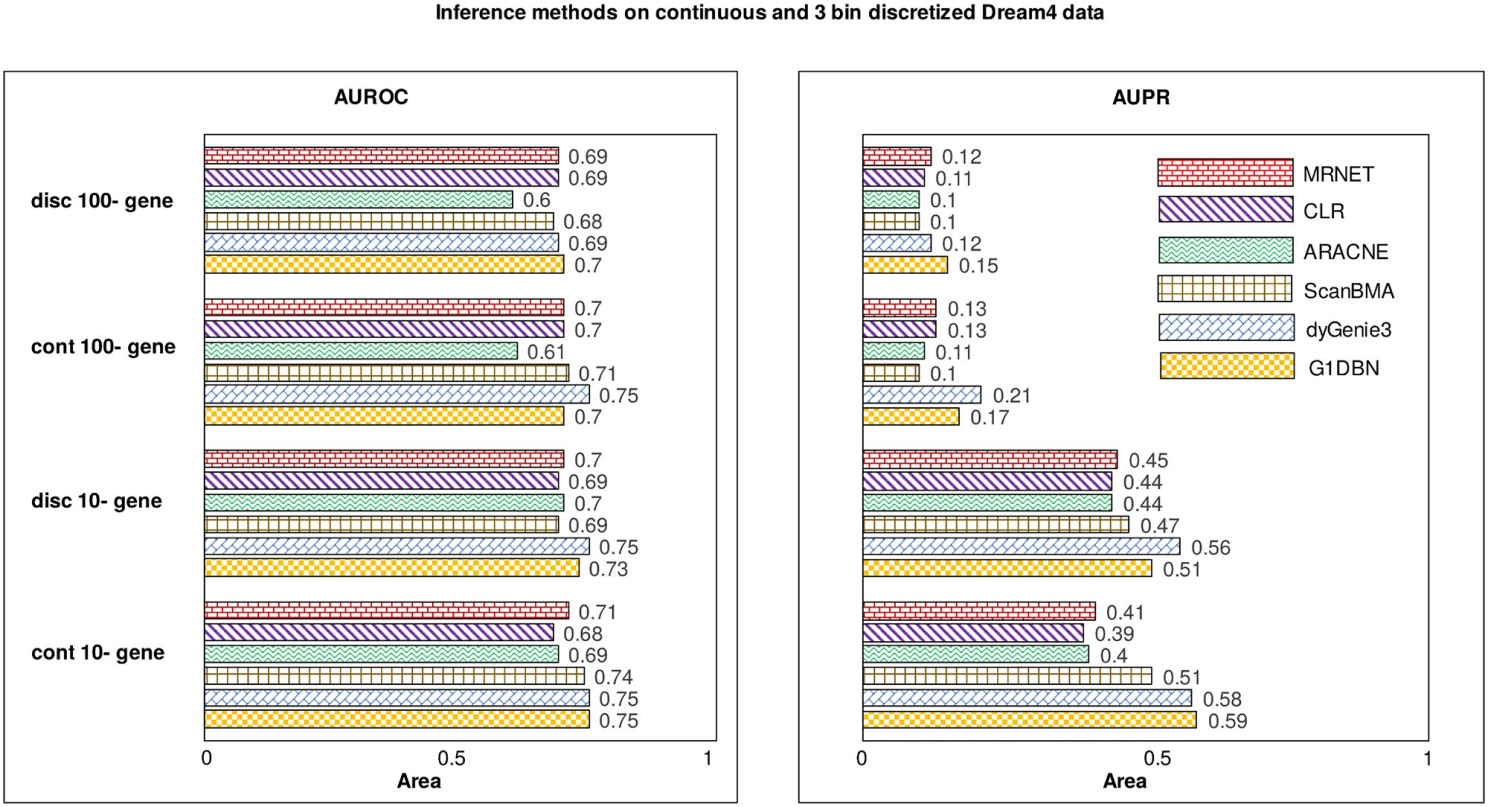
Performance comparison of the inference methods on continuous and discretized DREAM4 data. The bar plot shows the evaluation of inference methods using AUROC and AUPR metric. The only method affected much by discretization is the dynGenie3.

To examine the impact of data discretization on the performance of inference methods, the bar plot in Fig 5 compares the average AUROC and AUPR obtained on continuous as well as discretized DREAM4 data sets. Note that since in the case of discretized data sets, G1DBN and dynGenie3 achieved the best results on K-means data while scanBMA and mutual information-based methods performed their best on EFD data, here in the figure we have compared their performance on the continuous data with the discretized data on which they perform their best. On 100-gene data, it is seen that the performance of the dynGenie3 method dropped drastically on the discretized data with a decrease in performance of around 6% AUROC and 9% AUPR. The performance of all other methods is not impacted much, and only a drop of 1–2% is noticed in AUROC and AUPR. On 10-gene data, the accuracy of scanBMA is seen to be reduced by ∼4% AUROC and AUPR, and in the case of G1DBN, it is reduced by 8% AUPR. Since dynGenie3 and G1DBN, both are regression-based techniques, they work best with continuous data and hence seen the major drop in performance on discretized data. Though on 100-gene data, the performance of the G1DBN method is decreased by only 2% AUPR. Thus, the G1DBN is likely to perform well on discretized data if a large number of time series is available. Mutual information methods are seen to be the most unaffected by the discretization. Thus, though information loss is incurred by data discretization, most of the methods do not seem to be affected much by it.

### Real gene expression data sets

We applied the PEPN-GRN variant methods and the G1DBN method to infer the real network of organism *Escherichia coli*. *E. coli* is a well-studied organism in literature and its known biological knowledge can be used as an aid in network inference.

For the experiment, we used a sampled sub-network of *E. coli* having 236 genes, 257 edges, and 38 TFs. For the PEPN-GRN_v3 method, we sub-sampled another small network from the large network of 4511 genes for the training purpose. The train network contains 484 genes and 671 edges. For testing, we used the same sub-sampled network containing 236 genes and 257 edges. In the ground truth of the *E. coli* data set, edges do not contain a regulation sign. So, here, the training model considers all the edges present in the network as positive examples and all the non-edges as negative examples. Although the PEPN-GRN_v3 method generates two feature matrices (feature matrix contains four evidence probabilities of all gene pairs), one for activation and one for inhibition edges, here, for the model of edges vs. non-edges, both feature matrices will be equivalent. This is explained as follows: Suppose for an edge *g1*→*g2*, we have evidence probabilities for activation regulation (sign +) as {*p*1, *p*2, *p*3, *p*4} and similarly for inhibitory regulation (sign -), we have {*q*1, *q*2, *q*3, *q*4}. Suppose *g1*→*g2* exists in ground truth network with + sign. Therefore, the class label for *g1*→*g2* with + sign will be 1 (considered as positive example) while for edge *g1*→*g2* with—sign will be 0 (considered as negative example). Thus logistic regression model can be applied on both edges to separately learn weights for activation and inhibition edge. Now when we do not have information about the regulation sign of edges in the ground truth network, we cannot say which probability set to take as a positive example and which one to take as a negative example. We will basically have 8 features (evidence probabilities) for the edge *g1*→*g2*, out of which the first four features are of activating edge and the last four are of inhibiting edge. Now the first four features will be dependent on the last four features, e.g., if the production evidence probability (*p1*) for the activation edge is 0.7, for the inhibition edge, it will be 0.3 (*q1*), going by the logical rules of the PEPN-GRN method. Since in regression models, the features in training data of a model should be independent of each other, we can use any one of the evidence probabilities set as the feature set. Therefore for our experiment, we used a single 4-feature PEPN-GRN_v3 method for application on the *E. coli* data set.

The performance of the G1DBN and PEPN-GRN variant methods is evaluated on the sub-network and summarized in Table 6. In the G1DBN method, a threshold *α*1 is used to control the number of regulators for each target gene, and a threshold *α*2 is applied on the remaining edges to select the top significant edges having False Discovery Rate (FDR) ≤ 0.01. Here, thresholds *α*1 = 1 × 10^−10^ and *α*2 = 7.1 × 10^−3^ are chosen for continuous data and *α*1 = 1 × 10^−6^ and *α*2 = 7.7 × 10^−3^ for discretized data sets. To understand the approach to choose these thresholds, refer to S1 Appendix. Using the same assumptions, we have also used two thresholds for our PEPN-GRN methods to select the final set of edges. After obtaining the ranked edges using the PEPN-GRN method, the first *nReg* threshold is used to control the number of regulators for each target gene, and then threshold *th* is used to select the significant edges. We did not use heuristic approaches to select these thresholds. Here, the *nReg* threshold is chosen to be 3 to limit the number of regulator genes for each target gene to 3. For threshold *th*, different threshold values are tried and we chose *th* = 0.7, 0.7, and 0.6 for PEPN-GRN_v1, PEPN-GRN_v2 and PEPN-GRN_v3 respectively to select edges in top 200-300 range.

**Table 6:** Performance evaluation of the G1DBN and PEPN-GRN variant methods on the *E. coli* data set.

**(A) Number of inferred edges**		
**No. of inferred edges (True edges)**		
**Method**	**Continuous data**	
G1DBN	**142 (11)**	
	**EFD 3-bin data**	**K-means 3-bin data**
G1DBN	241 (18)	**333 (18)**
PEPN-GRN_v1	**209 (8)**	383 (10)
PEPN-GRN_v2	**341 (15)**	644 (18)
PEPN-GRN_v3	**259 (13)**	705 (21)
**(B) The average rank of random and true edges**		
**Average Rank (Random edges / True edges)**		
**Method**	**Continuous data**	
G1DBN	**78 / 33**	
	**EFD 3-bin data**	**K-means 3-bin data**
G1DBN	122 / 98	**162 / 81**
PEPN-GRN_v1	**99 / 104**	185 / 215
PEPN-GRN_v2	**174 / 123**	321 / 394
PEPN-GRN_v3	**121 / 105**	377 / 556

For the selection of significant edges, thresholds used in the G1DBN are: *α*1 = 1 × 10^−10^, *α*2 = 7.1 × 10^−3^ in case of continuous data and *α*1 = 1 × 10^−6^, *α*2 = 7.7 × 10^−3^ for discretized data sets. In PEPN-GRN variants, threshold *nReg* = 3 is used to limit the number of regulator genes for each target gene. Thresholds *th* used in the three variants are 0.7, 0.7, and 0.6 respectively to select top 300 edges. The table in (A) shows the number of inferred edges with the number of true edges in parentheses. The table in (B) shows the average rank of random and true edges in the inferred networks obtained by each inference method. TF information is used as background knowledge to restrict the number of edges.

Table 6(A) summarizes the number of inferred edges for each method. The performance of the G1DBN method is also compared for the continuous data as well as the discretized data. Using G1DBN, the number of inferred edges on the continuous data is 142 containing 11 true edges. On the K-means 3-bin discretized data set, the method retrieved 333 edges with 18 true edges. In the case of the PEPN-GRN variant methods, on the EFD 3-bin data, the PEPN-GRN_v1 inferred 209 edges with 8 true edges, PEPN-GRN_v2 obtained 341 edges with 15 true edges, and PEPN-GRN_v3 inferred 259 edges with 13 true edges. The performance of the methods is compared using the average rank metric (see Table 6(B)). The method’s performance is considered good when the average rank of random edges is higher, and that of true edges is lower. On the EFD 3-bin data set, the performance of G1DBN and PEPN-GRN_v3 is comparable: the average rank of 122/98 in the case of G1DBN and 121/105 in PEPN-GRN_v3. On the K-means 3-bin data set, G1DBN performed better with the average rank of 162/81. In the case of K-means data set, the improvement is also due to the fact that more edges are inferred in this case and thus the average rank of random edges increased. Also, it has been observed that though the number of inferred edges for the methods lies in the 300s, the number of shared edges between the methods is not significantly large.

### Contribution of evidence types

**Fig 6:**
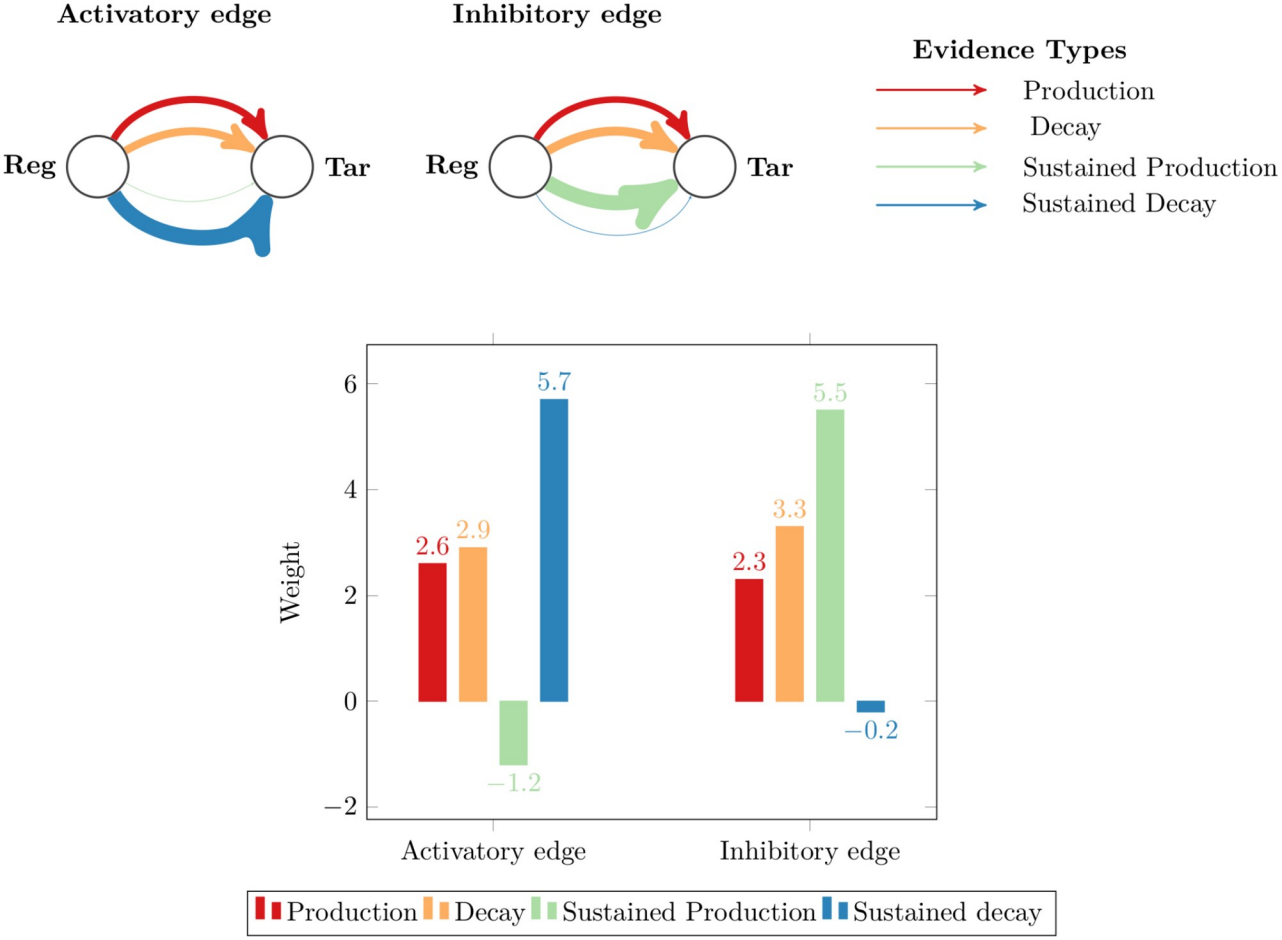
Contribution of evidence types in activatory and inhibitory edges. Four evidence types are shown as different color-coded links between a regulator and a target gene where the width of a link represents the learned weight for the corresponding evidence type. Below bar plot further shows the learned weights for both types of edges in case of EFD discretized 3-bin 100-gene DREAM4 data sets.

In the PEPN-GRN_v3 method, to understand the contribution of different evidence types in the activatory and inhibitory regulation of the gene, we analyzed the learned weights of the model in each EFD discretized DREAM4 data set (see Table 7(A)). The table shows for different EFD discretized data sets, the learned weights aggregated across all folds of 5-fold cross-validation. It is seen that all data sets except for the EFD 2-bin 10-gene data contain the same pattern of weight assignment to each evidence type. Looking at the signs of weights in activation edges, production, decay, and sustained decay evidence types increase the odds of the correct edges while the negative weight for sustained production evidence type decreases the odds of the correct edges. Similarly, for inhibition edges, production evidence, decay evidence, and sustained production evidence types obtained positive weights, thus contribute to increasing the odds of correct edges while sustained decay evidence decreases the odds. Among different evidence types, the sustained decay evidence contributes the most for the activation edges since it attains the highest weight, and sustained production evidence is more informative for inferring inhibition edges. The contribution of different evidence types in activation regulation and inhibition regulation of genes is depicted pictorially in Fig 6 where the four evidence types are shown as different color-coded links between regulator and target gene and the width of a link represents the learned weight of the corresponding evidence type. Higher weights for sustained production and sustained decay evidence types than for production and decay evidence types could also be because the instances of sustained production or sustained decay are seen to be much more than the switch from OFF to ON state for production and from ON to OFF state for the decay of gene expressions in the data.

**Table 7:** Learned weights [*w*_0_, *w*_1_, *w*_2_, *w*_3_, *w*_4_] in case of PEPN-GRN_v3 method on EFD discretized DREAM4 and *E. coli* data sets.

**(A) EFD discretized DREAM4 data**		
**Data**	**Activation edges**	**Inhibition edges**
2-bin 10-gene	[-3.116, 1.312, -0.212, 1.652, 3.076]	[-5.718, 2.382, 1.61, 4.31, 2.14]
2-bin 100-gene	[-6.422, 0.876, 2.908, -2.8, 10.912]	[-7.798, 3.536, 2.12, 11.968, -3.13]
3-bin 10-gene	[-3.776, 0.512, 2.65, -0.152, 5.486]	[-4.246, 3.932, 0.998, 4.584, -0.002]
3-bin 100-gene	[-4.49, 2.624, 2.94, -1.286, 5.768]	[-5.388, 2.386, 3.316, 5.558, -0.238]

**(B) EFD 3-bin discretized *E. coli* data**					
**Data**	**Activation edges**				
*E. coli*	-1.71	2.02	-2.9	2.43	-1.199

Here *w*_0_ is bias, weight *w*_1_ corresponds to production evidence, *w*_2_ corresponds to decay evidence, *w*_3_ corresponds to sustained production evidence, and *w*_4_ corresponds to sustained decay evidence. Table (A) shows the learned weights averaged across 5-fold cross validation in case of EFD discretized DREAM4 data sets. Table (B) shows the learned weights for activation edges in case of EFD discretized *E. coli* data set.

The contribution of evidence types can be associated with the underlying relationship shared by regulators and target genes. Yu et al. [51] have shown that the single input motifs exhibit time-shifted and inverted time-shifted relationships with their target genes in the *S. cerevisiae* gene network. In some way, the learned weights reflect these relationships between regulators and their target genes. For instance, for activation edges, we assumed in the logical rule defined for the production evidence type that the TF is ON (1) at time *t*-1 while the target gene transitions from OFF (0) to ON (1) state between adjacent time points. This reflects the time-delay or time-shift in the regulatory response of a target gene. In the logical rule of sustained production evidence, we assumed that the TF is ON at time *t*-1 and the target gene is ON at both time points *t*-1 and *t*. Negative weights for this evidence type suggest that our assumption is wrong and TF should be OFF at time point *t*-1. This can be explained as an inverted time-shifted relationship between TF and target gene where, after a time-delay (explained by production evidence), the relationship got reversed. Similar can be said in the case of inhibition edges as there negative weights are obtained for sustained decay evidence type. However, we obtained different weights on the real *E. coli* network (see Table 7(B)). Learned weights for different evidence types reveal that production and sustained production evidence contribute more than decay and sustained decay evidence in the prediction of edges. It should be noted that in *E. coli* data set, the knowledge of the regulation sign in the ground truth network is missing. So, the learned weights for the *E. coli* data set reveal the contribution of evidence types for the regulation of gene expression of a target gene by a regulator gene.

## Discussion and conclusions

In this paper, we proposed a probabilistic inference approach, *PEPN-GRN* for gene regulatory networks from noisy expression data sets. The approach is designed to work on discretized time series data sets. The novelty of the proposed approach lies in the four evidence types used for the inference of potential regulators for each target gene. We extended the earlier work done using the PN-based approaches (like Durzinsky et al. [32]) where only one evidence type related to the production of the gene expression is used for the inference of potential regulators for a target gene.

The paper presents three variants of the proposed PEPN-GRN inference approach which differ mainly by the way the different evidence probabilities for an inferred edge are aggregated for score computation. The performance of the variants is evaluated on benchmark *in silico* data from the DREAM4 challenge and real expression data of organism *E. coli* taken from the DREAM5 challenge. The experiments show that out of the three variants of the PEPN-GRN approach, PEPN-GRN_v3 performs the best followed by PEPN-GRN_v1 and then PEPN-GRN_v2. The PEPN-GRN_v3 is competitive with other inference methods as well since it outperforms all methods in the case of the DREAM4 data set. The second best performing method is the G1DBN method. On real data set, the performance comparison of two leading methods—the PEPN-GRN_v3 and the G1DBN method—reveals that among the top ≈300 inferred edges, both methods infer a small number of true edges (<20), and the number of shared edges by both methods is not large.

The inference methods (other than the PEPN-GRN method) considered in the paper infer the directed edges for a given data set. The PEPN-GRN approach extends the results by inferring directed edges along with their regulation signs. In PEPN-GRN_v3, the knowledge of regulation sign of the inferred edges and ground truth edges is used in learning weights of evidence types for activation and inhibition edges. The learned weights in the case of the DREAM4 data set help us understand the time-shifted and inverted time-shifted relationship between regulator and target gene. Thus, different evidence types and the knowledge of regulation sign of edges is used to explain the interplay between regulator and target gene at different time points, providing an insight into the functional understanding of the GRNs.

To examine the impact of discretization on inference methods other than the PEPN-GRN, the performance is compared on continuous as well as discretized DREAM4 data sets. The comparative results reveal that not much information loss is incurred by data discretization, and the methods still achieve good accuracy except for the regression-based methods like dynGenie3 and G1DBN. Though the drop in the performance of the G1DBN method is seen on 10-gene networks, the method performed well on 100-gene networks. Thus, the dynGenie3 method is the one most affected by discretization and hence not suitable to use with discretized data sets.

The application of the PEPN-GRN variant methods on real gene expression data set infers a large number of false positives, along with a few true positives. The performance of the G1DBN method on both continuous and discretized real data set is also quite similar. The results, however, are not surprising, and even the top-performing methods in the DREAM5 challenge that worked well on *in silico* networks did not achieve good performance on the *E. coli* data sets [6]. Thus, it requires us to come up with more sophisticated techniques that can include various aspects of the biological subsystems such as scale-free nature, structural properties, motifs, environmental factors, etc. that affect the interactions inside the cell. The current framework of the PEPN-GRN method allows for the addition of prior knowledge to some extent, like setting a maximum possible number of candidate regulators for each gene, and setting which genes can or cannot play the role of a regulator in a particular network. However, adding more domain-specific knowledge to the method would likely be helpful to predict the network more accurately. The Logic Guarded Transition System (LGTS) modeling framework which is a generalized form of an extended Petri net model, facilitates the incorporation of domain-specific knowledge in the form of logical rules. Using the existing knowledge of the subsystem not only helps in accurately inferring the network but also aids in gaining new insights about the dynamics. Thus, adapting the PEPN-GRN approach into the LGTS framework in future work would likely increase the usefulness of the inference approach.

## Supporting information

S1 Appendix Application of other inference methods on the DREAM4 data sets. (PDF)

## References

[1] Stark J, Brewer D, Barenco M, Tomescu D, Callard R, Hubank M. Reconstructing gene networks: what are the limits? Biochemical Society Transactions. 2003;31(6):1519–1525. 10.1042/bst0311519 14641103

[2] Marbach D, Prill RJ, Schaffter T, Mattiussi C, Floreano D, Stolovitzky G. Revealing strengths and weaknesses of methods for gene network inference. Proceedings of the National Academy of Sciences. 2010;107(14):6286–6291. 10.1073/pnas.0913357107 20308593 PMC2851985

[3] Markowetz F, Spang R. Inferring cellular networks—A review. BMC Bioinformatics. 2007;8(6). 10.1186/1471-2105-8-S6-S5 17903286 PMC1995541

[4] Bansal M, Belcastro V, Ambesi-Impiombato A, Di Bernardo D. How to infer gene networks from expression profiles. Molecular Systems Biology. 2007;3. 10.1038/msb4100120 17299415 PMC1828749

[5] Schlitt T, Brazma A. Current approaches to gene regulatory network modelling. BMC Bioinformatics. 2007;8(6). 10.1186/1471-2105-8-S6-S9 17903290 PMC1995542

[6] Marbach D, Costello JC, Küffner R, Vega N, Prill RJ, Camacho DM, et al. Wisdom of crowds for robust gene network inference. Nature Methods. 2012;9:796–804. 10.1038/nmeth.2016 22796662 PMC3512113

[7] Karlebach G, Shamir R. Modelling and analysis of gene regulatory networks. Nature Reviews Molecular Cell Biology. 2008;9(10):770–780. 10.1038/nrm2503 18797474

[8] Van Someren E, F A Wessels L, Backer E, Reinders M. Genetic Network Modeling. Pharmacogenomics. 2002;3:507–25. 10.1517/14622416.3.4.507 12164774

[9] Stolovitzky G, Prill RJ, Califano A. Lessons from the DREAM2 challenges. Annals of the New York Academy of Sciences. 2009;1158:159–195. 10.1111/j.1749-6632.2009.04497.x 19348640

[10] Aalto A, Viitasaari L, Ilmonen P, Mombaerts L, Gonçalves J. Gene regulatory network inference from sparsely sampled noisy data. Nature Communications. 2020;11(1). 10.1038/s41467-020-17217-1 32661225 PMC7359369

[11] Liu F, Zhang SW, Guo WF, Wei ZG, Chen L. Inference of Gene Regulatory Network Based on Local Bayesian Networks. PLOS Computational Biology. 2016;12(8):1–17. 10.1371/journal.pcbi.1005024 27479082 PMC4968793

[12] Mohan K, London P, Fazel M, Witten D, Lee SI. Node-based learning of multiple Gaussian graphical models. Journal of Machine Learning Research. 2014;15(1):445–488. 25309137 PMC4193819

[13] Rubiolo M, Milone DH, Stegmayer G. Extreme learning machines for reverse engineering of gene regulatory networks from expression time series. Bioinformatics. 2018;34(7):1253–1260. 10.1093/bioinformatics/btx730 29182723

[14] Castro JC, Valdés I, Gonzalez-García LN, et al. Gene regulatory networks on transfer entropy (GRNTE): a novel approach to reconstruct gene regulatory interactions applied to a case study for the plant pathogen *Phytophthora infestans*. Theoretical Biology and Medical Modelling. 2019;16(7). 10.1186/s12976-019-0103-7 30961611 PMC6454757

[15] Huynh-Thu VA, Sanguinetti G. In: Gene Regulatory Network Inference: An Introductory Survey. New York, NY: Springer New York; 2019. p. 1–23. Available from: 10.1007/978-1-4939-8882-2_1. 30547394

[16] Banf M, Rhee SY. Computational inference of gene regulatory networks: Approaches, limitations and opportunities. Biochim Biophys Acta Gene Regul Mech. 2017;1860(1):41–52. 10.1016/j.bbagrm.2016.09.003 27641093

[17] Huynh-Thu VA, Geurts P. DynGENIE3: Dynamical GENIE3 for the inference of gene networks from time series expression data. Scientific Reports. 2018;8(1):3384. 10.1038/s41598-018-21715-0 29467401 PMC5821733

[18] Palsson BO. Systems Biology: Properties of Reconstructed Networks. New York, USA: Cambridge University Press; 2006.

[19] Potapov AP. Signal Transduction and Gene Regulation Networks. In: Analysis of Biological Networks. John Wiley & Sons, Ltd; 2007. p. 181–206. Available from: https://onlinelibrary.wiley.com/doi/abs/10.1002/9780470253489.ch8.

[20] Klipp E. Systems Biology In Practice: Concepts, Implementation And Application. Wiley-VCH, Weinheim; 2005.

[21] Thomas R, Kaufman M. Multistationarity, the basis of cell differentiation and memory. II. Logical analysis of regulatory networks in terms of feedback circuits. Chaos: An Interdisciplinary Journal of Nonlinear Science. 2001;11(1):180–195. 10.1063/1.1349893 12779452

[22] Glass L, Kauffman SA. The logical analysis of continuous, non-linear biochemical control networks. Journal of Theoretical Biology. 1973;39(1):103–129. 10.1016/0022-5193(73)90208-7 4741704

[23] Ito S, Ichinose T, Shimakawa M, Izumi N, Hagihara S, Yonezaki N. Qualitative analysis of gene regulatory networks by temporal logic. Theoretical Computer Science. 2015;594:151–179. 10.1016/j.tcs.2015.06.017

[24] Schaub MA, Henzinger TA, Fisher J. Qualitative networks: A symbolic approach to analyze biological signaling networks. BMC Systems Biology. 2007;1. 10.1186/1752-0509-1-4 17408511 PMC1839894

[25] Küffner R, Petri T, Windhager L, Zimmer R. Petri Nets with Fuzzy Logic (PNFL): reverse engineering and parametrization. PLOS ONE. 2010;5(9):1–10. 10.1371/journal.pone.0012807 20862218 PMC2942832

[26] Marbach D, Schaffter T, Mattiussi C, Floreano D. Generating realistic in silico gene networks for performance assessment of reverse engineering methods. Journal of computational biology. 2009;16(2):229–239. 10.1089/cmb.2008.09TT 19183003

[27] Prill RJ, Marbach D, Saez-Rodriguez J, Sorger PK, Alexopoulos LG, Xue X, et al. Towards a rigorous assessment of systems biology models: The DREAM3 challenges. PLOS ONE. 2010;5(2):1–18. 10.1371/journal.pone.0009202 20186320 PMC2826397

[28] David R, Alla H. Discrete, Continuous, and Hybrid Petri Nets. Springer; 2005.

[29] Murata T. Petri nets: Properties, analysis and applications. Proceedings of the IEEE. 1989;77(4):541–580. 10.1109/5.24143

[30] Petri CA. Kommunikation mit Automaten. PhD thesis. Universität Hamburg; 1962.

[31] Durzinsky M, Wagler A, Weismantel R. A combinatorial approach to reconstruct Petri nets from experimental data. In: Computational Methods in Systems Biology. vol. 5307 of Lecture Notes in Computer Science. Berlin, Heidelberg: Springer; 2008. p. 328–346. Available from: 10.1007/978-3-540-88562-7_23.

[32] Durzinsky M, Wagler A, Marwan W. Reconstruction of extended Petri nets from time series data and its application to signal transduction and to gene regulatory networks. BMC Systems Biology. 2011;5(1). 10.1186/1752-0509-5-113 21762503 PMC3161898

[33] Durzinsky M, Marwan W, Wagler A. Reconstruction of extended Petri nets from time series data by using logical control functions. Journal of Mathematical Biology. 2013;66(1):203–223. 10.1007/s00285-012-0511-3 22302473

[34] Srinivasan A, Bain M. Knowledge-guided identification of Petri net models of large biological systems. In: Inductive Logic Programming. vol. 7207 of Lecture Notes in Computer Science. Berlin, Heidelberg: Springer; 2012. p. 317–331. Available from: 10.1007/978-3-642-31951-8_27.

[35] Vatsa D, Agarwal S, Srinivasan A. Learning transition models of biological regulatory and signaling networks from noisy data. In: Proceedings of the 3rd IKDD Conference on Data Science, 2016. CODS’16. New York, USA: ACM; 2016. p. 9:1–9:6. Available from: http://doi.acm.org/10.1145/2888451.2888469.

[36] Srinivasan A, Bain M, Vatsa D, Agarwal S. Identification of transition models of biological systems in the presence of transition noise. In: Inoue K, Ohwada H, Yamamoto A, editors. Inductive Logic Programming. Cham: Springer International Publishing; 2016. p. 200–214.

[37] Chawla NV, Bowyer KW, Hall LO, Kegelmeyer WP. SMOTE: Synthetic Minority Over-sampling Technique. Journal of Artificial Intelligence Research. 2002;16(1):321–357. 10.1613/jair.953

[38] Li Y, Liu L, Bai X, Cai H, Ji W, Guo D, et al. Comparative study of discretization methods of microarray data for inferring transcriptional regulatory networks. BMC Bioinformatics. 2010;11(1):520. 10.1186/1471-2105-11-520 20955620 PMC2967565

[39] Catlett J. On changing continuous attributes into ordered discrete attributes. In: Kodratoff Y, editor. Machine Learning—EWSL-91. Springer, Berlin, Heidelberg; 1991. p. 164–178.

[40] Kerber R. ChiMerge: Discretization of numeric attributes. In: Proceedings of the Tenth National Conference on Artificial Intelligence. AAAI’92. AAAI Press; 1992. p. 123–128. Available from: http://dl.acm.org/citation.cfm?id=1867135.1867154.

[41] Dougherty J, Kohavi R, Sahami M. Supervised and unsupervised discretization of continuous features. In: Prieditis A, Russell S, editors. Machine Learning Proceedings 1995. San Francisco (CA): Morgan Kaufmann; 1995. p. 194–202. Available from: http://www.sciencedirect.com/science/article/pii/B9781558603776500323.

[42] MacQueen J. Some methods for classification and analysis of multivariate observations. In: Proceedings of the Fifth Berkeley Symposium on Mathematical Statistics and Probability, Volume 1: Statistics. Berkeley, California: University of California Press; 1967. p. 281–297. Available from: https://projecteuclid.org/euclid.bsmsp/1200512992.

[43] Cokelaer T, Bansal M, Bare C, et al. DREAMTools: a Python package for scoring collaborative challenges. F1000Research. 2015;4 (1030). 10.12688/f1000research.7118.2 27134723 PMC4837986

[44] Young WC, Raftery AE, Yeung KY. Fast Bayesian inference for gene regulatory networks using ScanBMA. BMC Systems Biology. 2014;8(1):47. 10.1186/1752-0509-8-47 24742092 PMC4006459

[45] Lèbre S. Inferring dynamic genetic networks with low order independencies. Statistical applications in genetics and molecular biology. 2009;8(Article 9). 19222392 10.2202/1544-6115.1294

[46] Meyer PE, Lafitte F, Bontempi G. minet: A R/Bioconductor package for inferring large transcriptional networks using mutual information. BMC Bioinformatics. 2008;9(1):461. 10.1186/1471-2105-9-461 18959772 PMC2630331

[47] G1DBN package;. https://CRAN.R-project.org/package=G1DBN.

[48] networkBMA package;. https://bioconductor.org/packages/release/bioc/html/networkBMA.html.

[49] MINET package;. https://bioconductor.org/packages/release/bioc/html/minet.html.

[50] dynGENIE3 package;. https://github.com/vahuynh/dynGENIE3/tree/master/dynGENIE3_R_C_wrapper.

[51] Yu H, Luscombe NM, Qian J, Gerstein M. Genomic analysis of gene expression relationships in transcriptional regulatory networks. Trends in Genetics. 2003;19(8):422–427. 10.1016/S0168-9525(03)00175-6 12902159

