# Supplementary material for: PEPN-GRN: A Petri net-based approach for the inference of gene regulatory networks from noisy gene expression data
Source: PLoS One. 2021 May 14;16(5):e0251666. doi: 10.1371/journal.pone.0251666 (PMC8121333; doi:10.1371/journal.pone.0251666)
Supplement: S1 Appendix — (PDF) [file pone.0251666.s001.pdf]

PEPN-GRN: A Petri net-based approach for the inference of gene regulatory networks from noisy gene expression data

S1 Appendix. Application of other inference methods on the DREAM4 data sets

In the experiment on the DREAM4 *in silico* networks, apart from the PEPN-GRN variant methods, other network inference methods used are Bayesian-based scanBMA, regression-based DBN inference model G1DBN, Random Forest-based dynGENIE3, and mutual information-based ARACNE, CLR, and MRNET. Among these, mutual information-based methods, i.e., ARACNE, CLR, and MRNET, reconstruct networks using a steady-state data set. Therefore, we used a *time-shift* technique (as discussed in [1]) on the DREAM4 time series data to generate the time-lagged time series data and then obtained results.

The DynGenie3 method, on the other hand, works only on continuous time series data and can not work on discretized data sets. Thus, to evaluate its performance on discretized data, a little amount of white Gaussian noise is added to the discretized data to make it continuous, and then the experiment is performed. The procedure for the addition of noise is as follows: The noise $\mathcal{N}(0, s)$ with mean $\mu = 0$ and standard deviation s is added to the discretized data set D of size $m \times n$. We have computed the standard deviation s as:

$$s = \sigma(D * 10^\wedge(-SNR/20))$$

where signal-to-noise ratio SNR is taken to be 50 in our experiment. The noise is computed as:

$$noise = \mu + random(m, n) * s$$

where $random(m{,}n)$ is a matrix of size $m \times n$ of normally distributed random numbers.

The G1DBN method is a two-step approach that uses an auto-regressive model to estimate a score matrix of size $p \times p$ using a time series data of p genes. The Step 1 infers a score matrix that describes first-order dependencies

of gene pairs. A threshold $\alpha 1$ is applied on the score matrix to select the gene pairs such that the density of target gene having 0 or 1 regulator genes is much larger than that of target genes with 2 or more regulators. On the score matrix of the selected gene pairs, Step 2 is applied, which generates a score matrix describing full-order dependencies of selected genes. Applying another threshold $\alpha 2$ on the second score matrix returns the final set of edges. The threshold $\alpha 2$ is chosen using the Benjamini-Hochberg approach [2] to select the significant edges such that they contain False Discovery Rate (FDR) ≤ 0.01. Thus, threshold $\alpha 1$ helps in dimensionality reduction and is based on the assumption that in gene networks, most of the target genes contain only a few TFs. Threshold $\alpha 2$ is then used to select the final set of significant edges. For the application of the G1DBN method on the DREAM4 data, threshold $\alpha 1 = 1$ is used since the DREAM4 data sets contain a small number of genes (10 and 100), and thus no dimensionality reduction is required. Therefore all the edges except self-edges are considered here.

References

1. Hempel S, Koseska A, Nikoloski Z, Kurths J. Unraveling gene regulatory networks from time-resolved gene expression data – a measures comparison study. BMC Bioinformatics. 2011;12(1):292. doi:10.1186/1471-2105-12-292.

2. Benjamini Y, Hochberg Y. Controlling the False Discovery Rate: A Practical and Powerful Approach to Multiple Testing. Journal of the Royal Statistical Society Series B (Methodological). 1995;57(1):289–300.
